# Packaging Glasses: From Containers to Encapsulation *Composition, Performance, and Sustainability Pathways*

**DOI:** 10.3390/ma19030506

**Published:** 2026-01-27

**Authors:** Leonardo Pagnotta

**Affiliations:** Department of Mechanical, Energy and Management Engineering, University of Calabria, 87036 Rende, Italy; leonardo.pagnotta@unical.it

**Keywords:** packaging glass, soda–lime glass, borosilicate glass, aluminosilicate glass, recycled/cullet-rich glass, functional and electronic encapsulation, ion exchange strengthening, ALD coatings, sustainability, circular economy

## Abstract

**Highlights:**

**What are the main findings?**

- Identification and classification of the main packaging-glass families.
- Correlation between glass composition, processing routes, and functional performance.
- Quantitative comparison of circularity indicators, including cullet content, energy demand, CO_2_ footprint, and regulatory compliance.
- Systematic overview of technological innovations in packaging glass, including ion exchange, ALD coatings, and lightweight forming.

**What is the implication of the main finding?**

- Glass is framed as a chemically inert and permanently recyclable packaging material.
- The scope of packaging glass is extended from conventional containers to hermetic and electronic encapsulation systems.
- The analysis supports the role of glass within decarbonized and traceable packaging supply chains.

**Abstract:**

This review synthesizes four decades of scientific and industrial developments in packaging glass, integrating structural, technological, and sustainability perspectives. Glass remains the benchmark material for inert, transparent, and fully recyclable containment, yet its scope has expanded from conventional bottles and vials to advanced functional and electronic encapsulation. Packaging glasses are classified into five main families—soda–lime, borosilicate, aluminosilicate, recycled (cullet-rich), and functional/electronic—and compared across key domains: mechanical, thermal, chemical, optical, barrier, and hermetic. Quantitative tables and normalized diagrams illustrate how compositional and processing trends govern structure, processability, and performance. Advances in forming, surface engineering, and melting practice are analyzed for their contributions to lightweighting, durability, and decarbonization. Sustainability is addressed through cullet utilization, energy demand, life-cycle indicators, and regulatory alignment, defining pathways toward circular and low-carbon production. Overall, packaging glass emerges as a circular, chemically stable, and traceable material system, while advances in high-integrity glass formulations now support hermetic encapsulation for diagnostic, electronic, and energy devices.

## 1. Introduction

Glass has long been one of the most durable and trusted materials in human material culture, and it remains a cornerstone of modern packaging. Its well-established use for beverages and preserved foods—documented for more than a century—derives from its gas impermeability, chemical inertness, and exceptional long-term storage stability [1]. These attributes, together with resistance to moisture, oxygen, and external contaminants, have preserved its reputation as a high-quality packaging material [2,3], a role highlighted across historical assessments of packaging materials [2,3,4].

Although challenged by lighter and cheaper polymeric alternatives [5], glass continues to represent the benchmark for safety, performance, and recyclability. Its negligible interaction with contents [6,7,8,9] and its closed-loop recyclability sustain its competitiveness within an evolving materials landscape. Industrial innovations—most notably the narrow-neck press-and-blow process—have enabled significant lightweighting while maintaining mechanical integrity [7,8,10]. In parallel, high recycling rates exceeding 90% in countries such as Sweden and Switzerland [8,11] and the use of up to 80% cullet in furnace feedstocks [11,12,13] significantly reduce energy demand and virgin-raw-material consumption. Life-cycle assessments consistently show that efficient collection and reuse loops allow glass to attain lower environmental impacts than polymeric or metallic alternatives [14,15].

Progress in materials science has refined glass as a functional engineering material. Adjustments of network formers and modifiers—Na_2_O, CaO, MgO, and Al_2_O_3_—provide precise control of viscosity, formability, and chemical durability [13]. At the atomic scale, computational and spectroscopic studies have deepened understanding of dopant behaviour, hydroxyl mobility, and network stability [16]. Surface-engineering approaches, including sol–gel nanocoatings and ion-exchange strengthening, have improved scratch resistance, barrier performance, and flexural strength [17,18], while long-term chemical stability and minimal element release continue to support glass in food, biomedical, and pharmaceutical applications [9,19].

In parallel, sustainability-oriented innovation has focused on decarbonizing production through hybrid-electric and oxy-fuel furnaces and on maximizing recycled content [20,21]. These initiatives—coordinated within FEVE (European Container Glass Federation)—align with UN Sustainable Development Goals 12 and 13 and reinforce glass as a climate-responsible manufacturing system. Regulatory frameworks such as Regulation (EU) 2023/2006 and FDA 21 CFR 175.300 ensure traceability and compliance with migration limits [22], while consumer studies consistently confirm glass as a safe, transparent, and environmentally responsible choice.

Beyond conventional containers, glass is increasingly engineered as a multifunctional packaging platform for diagnostic, electronic, and energy technologies. Microstructured borosilicate and fused-silica substrates enable microfluidic devices and biosensors [23,24], while low-alkali aluminosilicates and hybrid boro-aluminosilicates provide dielectric insulation, thermal-expansion matching, and long-term hermeticity in photovoltaic modules, LEDs, and MEMS [25,26,27]. For hazardous or extreme environments, boro–aluminosilicate hybrids ensure corrosion resistance and thermal stability [26,28].

Compared with other major packaging materials, glass occupies a distinctive position [2,3,4]. Unlike polymers, it provides an absolute barrier to gases and vapours, exhibits negligible interaction with contents, and does not suffer from ageing or additive migration [1,6,7,8,9]. Relative to metals, glass is chemically inert, corrosion-free in contact with food and pharmaceutical products, and intrinsically transparent, enabling direct visual inspection [9,19,22]. Compared with paper-based systems, glass offers complete hermeticity and long-term stability without the need for multilayer coatings or functional additives [2,3,4,14,15]. These attributes, combined with permanent recyclability without loss of properties, define glass as a benchmark material for safe, durable, and circular packaging applications [11,12,13,14,15,20,21].

These developments motivate the inclusion of a fifth family—functional and electronic packaging glasses—alongside traditional soda–lime, borosilicate, aluminosilicate, and recycled compositions. In this context, a comprehensive and updated synthesis is needed to connect historical evolution, compositional design, processing routes, functional performance, and sustainability targets within a unified analytical framework. This review addresses this need by combining qualitative insight with quantitative comparisons, using normalized performance diagrams to evaluate how compositional trends influence structure, manufacturability, and long-term reliability across contemporary packaging-glass families.

In the remainder of the manuscript, packaging glasses are organized into five families based on composition and application domain: soda–lime (mass container glass), borosilicate (pharmaceutical and laboratory packaging), aluminosilicate (high-strength and chemically strengthened formats), recycled/cullet-rich (high-recycled-content container streams), and functional/electronic systems (substrates, seals, and glass–ceramics for hermetic and dielectric encapsulation). Partial overlaps are discussed where they arise from shared base chemistries deployed under different processing and application regimes.

**Figure 1:**
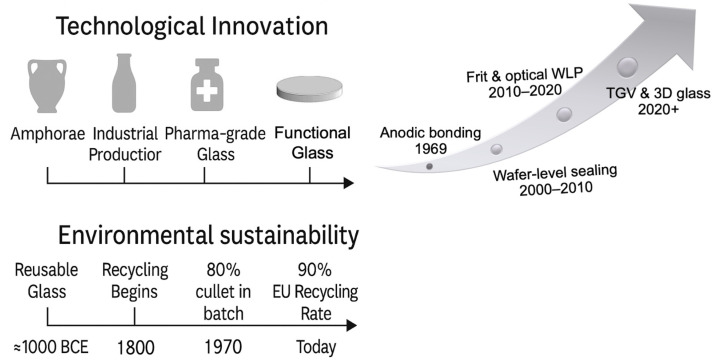
Technological and environmental evolution of packaging glass. Top: Technological trajectory from amphorae and early industrial bottles to pharma-grade containers and functional glass platforms for electronic and photonic packaging. Bottom: Environmental trajectory from reusable systems to organized recycling schemes, high-cullet batches, and current EU recycling rates around 90%. The curved arrow schematically indicates the extension of packaging glass toward advanced TGV-based and other functional architectures.

**Figure 2:**
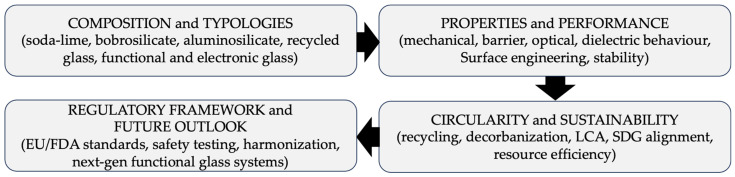
Conceptual structure of the review: Interrelation among composition and typologies, properties and performance, circularity and sustainability, and regulatory framework and future outlook in packaging glass. The diagram summarizes the logical organization of the review and highlights the progressive link from material design to system-level sustainability and regulation.

Figure 1 and Figure 2 outline the historical, environmental, and conceptual architecture guiding the discussion that follows.

Throughout the manuscript, each packaging-glass family is discussed using a consistent analytical framework addressing composition, processing routes, key functional properties, surface interactions, and packaging applications, in order to facilitate comparison across material classes.

### 1.1. Historical and Technological Evolution of Packaging Glass

The origins of glassmaking extend deep into antiquity, with early forms of glass appearing as far back as 7000 BC and more structured production emerging in Egypt around 1500 BC [29]. The first hollow containers—core-formed vessels produced in Egypt and Mesopotamia in the fifteenth century BC—introduced the concept of glass as a chemically stable container. A decisive technological shift occurred with the invention of the blowpipe in the Syro-Palestinian region around the first century BC, which enabled faster forming, thinner walls and a much broader range of vessel shapes [30,31]. Parallel developments during the Wei–Jin and Sui–Tang periods expanded early Eastern glassmaking traditions [32].

Through the medieval and early-modern eras, European production centres—particularly Venice and Murano—refined furnace practice and soda-ash formulations, giving rise to cristallo [33]. Compositional surveys by Brill (1999) [34] and Freestone (2023) [35] document the long transition from plant-ash glasses to low-alkali soda–lime formulations, which would later underpin industrial-scale container manufacturing.

The nineteenth and early twentieth centuries marked the shift from craft to mechanized production. Regenerative furnaces, continuous melting and the Owens automatic bottle-blowing machine (1903) opened the way to large-volume manufacturing [34,36]. By mid-century, individual-section (IS) machines [37], mould standardization, and improved annealing established a consistent industrial workflow, strengthening reliability and throughput [36].

Subsequent decades consolidated this industrial architecture. Forming evolved from blow–blow and press–blow processes to the narrow-neck press-and-blow (NNPB) approach, supported by servo-controlled gob delivery and improved mould-thermal management, enabling more uniform parisons and enhanced structural consistency [37,38]. Parallel advances in composition—such as adjustments in MgO, Al_2_O_3_ and Fe_2_O_3_ contents—expanded working ranges and improved chemical durability. Borosilicate systems matured as the reference for Type I pharmaceutical containers, combining low thermal expansion with exceptional hydrolytic stability [2,3].

From the 1990s onward, environmental priorities shaped manufacturing trajectories. Closed-loop production and high-purity cullet streams became structural elements of European container-glass operations [7,11], while cleaner melting technologies—hybrid-electric and oxy-fuel furnaces, oxygen enrichment, and batch preheating—reduced specific energy demand and CO_2_ intensity [20,21]. Life-cycle assessments reinforced these developments by demonstrating the advantages of high-recycling, short-haul systems [12,13,14,15]. Advances in spectroscopy, modelling and surface engineering further clarified how composition and nanostructure govern viscosity and durability [16,18].

In parallel with container production, glass increasingly assumed functional roles in electronic and photonic packaging. Electrostatic Si–glass bonding introduced by Wallis and Pomerantz (1969) [39]—and later clarified through studies on ionic migration, oxygen evolution, and thermomechanical behaviour [40]—enabled hermetic wafer-level sealing in MEMS. A complementary low-temperature route, based on Pb-free and low-alkali frits, matured through studies on rheology, wetting, and densification [41]. Optical packaging adopted transparent borosilicate caps with antireflective coatings and micromachined geometries [42,43].

After 2020, alkali-free aluminoborosilicate and borosilicate substrates supported fine-pitch metallization and through-glass-via (TGV) architectures [44,45,46], while low-loss borosilicates, Ca–SiO_3_ glass–ceramics, and lead–aluminosilicate systems addressed RF, thermal management, and passivation needs [47,48,49]. Recent applications demonstrated the use of glass interposers and TGV substrates in MEMS, MOEMS, RF, and sensor packaging [50,51].

Across this long trajectory—from early core-formed vessels to modern industrial bottles and multifunctional substrates—glass has evolved from a labour-intensive craft to both a pillar of contemporary packaging and a platform material enabling advanced technological architectures. Figure 1 summarizes the main historical and environmental milestones for both container and functional packaging glasses, complementing the conceptual framework shown in Figure 2.

### 1.2. Methodological Note: Scope and Selection Criteria

This review adopts the principles of transparency and structured synthesis promoted by PRISMA 2020, adapted to a mixed qualitative–quantitative approach. Its objective is to provide a traceable overview of packaging-glass materials by integrating structural, mechanical, environmental, and regulatory perspectives within a unified framework. The literature search, completed in October 2025, used Scopus, Web of Science, ScienceDirect, and Google Scholar as primary databases, supplemented by MDPI, SpringerLink, Elsevier, and institutional repositories such as the Corning Museum of Glass and The Metropolitan Museum of Art. The time window (1986–2025) spans both the historical development of container glass and contemporary studies on decarbonization, circularity, and functional encapsulation. Inclusion criteria targeted works providing technical or analytical content on composition, processing, performance, or sustainability across the five packaging-glass families: soda–lime, borosilicate, aluminosilicate, recycled (cullet-rich), and functional/electronic glasses. Exclusion criteria concerned items lacking methodological transparency or addressing glass types unrelated to packaging (e.g., display or fibre optics) unless their findings were directly applicable. Duplicates were screened manually and through database filters to ensure unique records per publication.

The search keywords included the following: packaging glass, soda–lime, borosilicate, aluminosilicate, recycled glass, cullet, functional glass, electronic packaging, hermetic sealing, ion exchange, ALD coatings, sustainability, circularity, life-cycle assessment, PPWR, and Green Deal. Peer-reviewed articles and conference proceedings were both considered when they provided validated data or industrially relevant benchmarks.

Each source was examined to extract data on composition, processing routes, and functional performance. Quantitative information—mechanical strength, optical transmittance, barrier metrics—was consolidated into comparative tables, while qualitative analyses were used to identify technological trends and sustainability pathways across the five glass families. This approach ensures chronological coherence and aligns with the objective of evaluating packaging glass as an evolving material system linking innovation, safety, and circularity.

**Figure 3:**
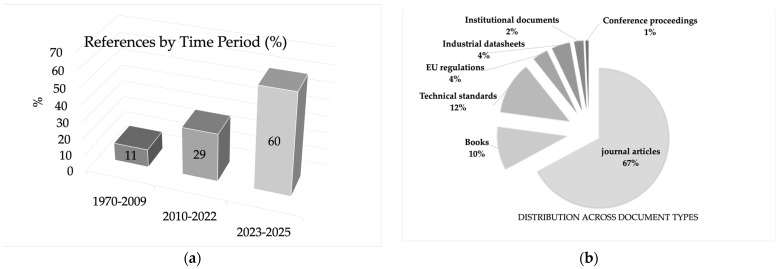
Overview of the 141 references used in this review: (**a**) Temporal distribution of the literature across three time periods: 1970–2009 (11%), 2010–2022 (29%) and 2023–2025 (60%). (**b**) Distribution by document type: peer-reviewed journal articles (67%), books and book chapters (10%), technical standards (12%), EU regulations (4%), industrial datasheets and corporate technical documents (4%), institutional technical documents (2%) and conference proceedings (1%). Percentages reflect the composition of the review corpus at the time the literature search was closed (October 2025).

The final reference set comprises 141 sources covering the period 1969–2025. Their temporal distribution is strongly skewed towards the most recent years (Figure 3a). Only 11% of the references were published between 1970 and 2009, while 29% fall in the intermediate window 2010–2022. The remaining 60% belong to 2023–2025, reflecting the rapid growth of work on decarbonised melting, cullet quality, advanced forming, and functional/electronic packaging glasses in the last three years.

In terms of document type, the corpus is dominated by peer-reviewed journal articles, which account for 67% of all references (Figure 3b). Books and book chapters represent 10%, and technical standards (ISO, EN, USP, Ph. Eur.) contribute a further 12%. EU regulations and policy documents, industrial datasheets and corporate technical reports, institutional technical documents (EPA, BREF, NGOs), and conference proceedings together make up the remaining 11%. This mix confirms that the review is mainly grounded in the primary scientific literature while also integrating normative, regulatory, and industrial sources that are essential for assessing compliance, circularity, and process performance.

## 2. Typologies and Compositions of Packaging Glass

To maintain a consistent interpretation of the material families discussed in this section, the distinct roles of the summary tables are clarified here. Table 1 introduces a functional classification of packaging-glass encapsulation systems, linking components, sealing modes, and typical material families. Conventional container-glass families—soda–lime, pharmacopeial borosilicate, aluminosilicate container glass, and cullet-rich recycled compositions—are summarized separately in Table 2 and are discussed in Section 2.1, Section 2.2, Section 2.3 and Section 2.4 for food, cosmetic, and pharmaceutical packaging applications. Table 3 compiles representative compositions and quantitative property ranges for functional and electronic packaging glasses used in microsystems and high-reliability assemblies.

The functional requirements of packaging—chemical inertness, thermal stability during filling and sterilization, mechanical robustness under transportation loads, gas impermeability, long-term product safety, and regulatory compliance—determine which glass compositions can be effectively used in different sectors. For this reason, the analysis of packaging glass cannot be separated from its compositional design and network structure: the nature and proportion of network formers, modifiers, and stabilizers directly influence durability, viscosity windows, forming behaviour, optical properties, and resistance to chemical or thermal stress during service.

**Figure 4:**
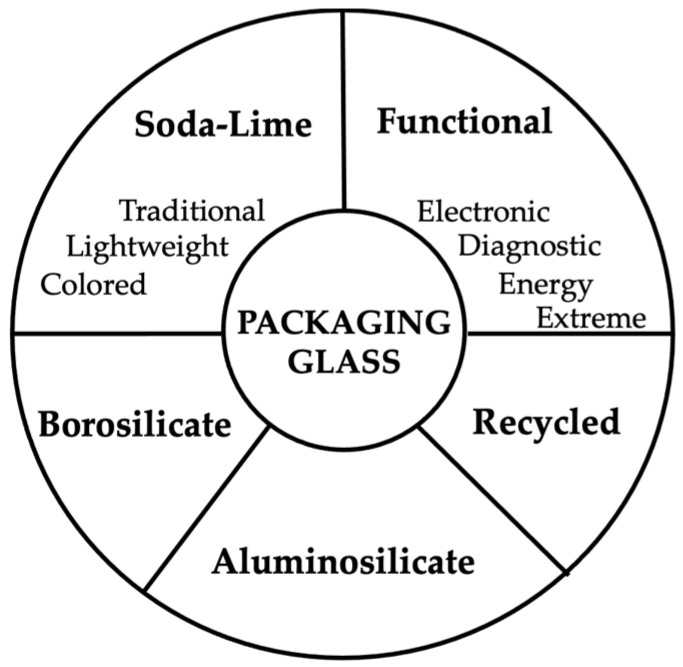
Classification of packaging-glass families. Schematic representation of the five main families of packaging glass—Soda–Lime, borosilicate, aluminosilicate, recycled (cullet-rich), and functional/electronic—grouping the glass types used in packaging applications and distinguishing conventional container compositions from functional/electronic systems.

Packaging glass can be grouped into five families—soda–lime, borosilicate, aluminosilicate, recycled (cullet-rich), and functional/electronic compositions—according to structural and technological criteria [3,10,12,25,26,52,53,54,55,56]. These five families, schematically classified in Figure 4, provide the comparative basis for the analysis developed in this section.

**Figure 5:**
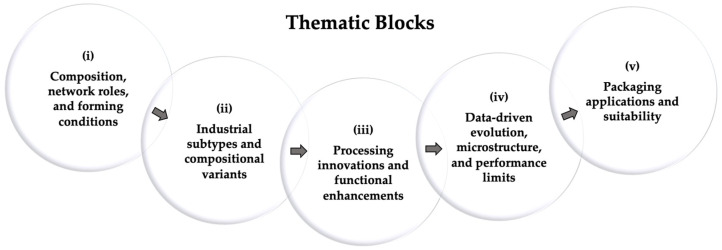
Analytical structure applied to all packaging-glass families. The diagram illustrates the five thematic blocks used consistently in Section 2.1, Section 2.2, Section 2.3, Section 2.4 and Section 2.5: (i) composition, network roles, and forming conditions; (ii) industrial subtypes and compositional variants; (iii) processing innovations and functional enhancements; (iv) data-driven evolution, microstructure, and performance limits; and (v) packaging applications and suitability.

To ensure a coherent structure across families, each of the following sections is articulated into five analytical blocks (Figure 5):

(i) Composition, network roles, and forming conditions.
(ii) Industrial subtypes and compositional variants.
(iii) Processing innovations and functional enhancements.
(iv) Data-driven evolution, microstructure, and performance limits.
(v) Packaging applications and suitability.

This framework enables a consistent comparison among glass families, linking composition, processing, and performance to their deployment in modern packaging.

**Table 1:** Representative glass components and their primary functions within packaging architectures. Each element fulfils structural and/or functional roles—hermetic sealing, dielectric insulation, optical transmission, or chemical containment—depending on composition and joining process. The listed glass families and processing routes correspond to the main technological classes discussed in this section.

Glass Component	Primary Function in Packaging	Typical Glass Families and Processes
Capping lids/ optical windows	Hermetic sealing, optical or IR transmission, mechanical protection of MEMS and sensors	Borosilicate (Pyrex, Borofloat), aluminosilicate, LAS glass–ceramics; anodic, frit, or laser bonding
Sealing layers/ bonding frits	Pb-free hermetic sealing, dielectric isolation, adhesion to metals or ceramics	Bi_2_O_3_–B_2_O_3_–ZnO, Ba–Zn–B_2_O_3_, phosphate–silicate frits; screen-printing, jet deposition, localized sintering
Substrates/ interposers (TGV)	Electrical insulation, vertical interconnection, dimensional stability	Alkali-free borosilicate or aluminoborosilicate; laser drilling, chemical etching, metallization, planarization
Feedthroughs/ frames/spacers	Mechanical alignment, electrical feedthrough, cavity definition	Borosilicate, aluminosilicate, glass–ceramic rings; diffusion or glass-to-metal bonding
Microfluidic chips/passivation layers	Chemical inertness, optical access, bio-compatibility, corrosion protection	Borosilicate–phosphate hybrids, ALD-coated aluminosilicates; wet etching, additive microfabrication
Glass–metal/ glass–ceramic joints	Long-term hermeticity and insulation in harsh environments	Borosilicate–aluminosilicate with ZrO_2_ or TiO_2_, LAS glass–ceramics; compression or diffusion sealing

**Table 2:** Technical comparison of the four main packaging glass families: soda–lime, Type I/III Borosilicate, Aluminosilicate, and recycled (cullet-rich) glass. The table summarizes typical compositions, physical and mechanical properties (density, Young’s modulus, tensile, compressive, and flexural strength, hardness, Poisson’s ratio), and thermal–chemical parameters (coefficient of thermal expansion, hydrolytic class, and optical/UV protection). Qualitative items describe recyclability, sustainability indicators, and main application sectors. References listed under each glass type correspond to the sources from which all data in that column were extracted or validated.

Parameter (Units)	Soda–Lime	Type I/III Borosilicate	Aluminosilicate	Recycled (Cullet-Rich)
	[3,13,57,58,59,60,61]	[3,6,46,48,52,54,55,56,62,63]	[6,17,25,27,52,53,55,64]	[12,13,21,56,60,61,65,66]
Typical composition (wt%)	SiO_2_ 70–74; Na_2_O 12–14; CaO 9–11; MgO 3–4; Al_2_O_3_ 1–2.	SiO_2_ 78–81; B_2_O_3_ 12–13; Na_2_O/K_2_O 4–5; Al_2_O_3_ 2–3.	SiO_2_ 73–77; Al_2_O_3_ 6–12; MgO/CaO 5–8; Na_2_O/K_2_O 3–5.	SiO_2_ 69–73; Na_2_O 12–14; CaO 8–10; MgO 3–4; Al_2_O_3_ 1–2; Fe_2_O_3_ 0.1–0.7; Cr_2_O_3_ ≤ 0.3 (from flint/amber/green cullet mixtures)
Density (g cm^−3^)	2.48–2.55	2.23–2.30	2.42–2.48	2.47–2.50 (till to ~2.55 for green cullet)
Young’s modulus (GPa)	70–72	61–65	70–75	70–76 (measured on recycled soda–lime glass, IET/4PB)
Tensile strength (MPa)	45–85	45–80	60–90	40–70 (≈70 flint → 55 amber → 45 green)
Compressive strength (MPa, typ.)	800–1000	900–1100	1000–1200	850–950 (≈−10% for green cullet)
Flexural strength (MPa)	60–150	70–130	90–150	43.7–47.7 (annealed cullet-based specimens)
Hardness (HV, Vickers)	540–580	530–560	600–650	≈540 ± 15 (≤10% variation)
Fracture toughness K_IC_ (MPa m^1/2^)	0.65–0.75	0.8–1	0.9–1.2	0.65–0.75 (nearly invariant)
Poisson’s ratio (–)	0.22–0.24	0.20–0.22	0.21–0.23	≈0.23 (± 0.01)
CTE (10^−6^ K^−1^, 20–300 °C)	8.5–9.0	3.2–3.4	3.8–4.2	8.5–8.8 (≈8.5 flint → 8.9 green)
Hydrolytic class (EU/Ph. Eur.)	II–III	I	I	II → I (for cullet > 60%; amber slightly more stable).
Optical/UV protection (qual.)	Clear/amber/green; amber or coatings for UV	High clarity; optional ALD/UV coatings	Colourless premium; coatings as needed	UV cut-off: ≈320 flint → 450 amber → 420 green nm.
Gas barrier (O_2_/H_2_O permeability)	Practically zero	Practically zero	Practically zero	Practically zero
Key characteristics (qual.)	High productivity; cost-efficient; good processability	Highest thermal/chemical stability; sterilizable	Highest stiffness/hardness; thin walls; premium look	Lowest footprint with high cullet; stable properties across remelts
Recyclability and cullet (qual.)	Fully recyclable; typical cullet 30–60%	Fully recyclable (specialized lines)	Fully recyclable (premium lines)	Fully recyclable; cullet 50–90%; quality depends on sorting and contaminants
Sustainability impact (LCA, indicative)	+10% cullet → ~3% energy ↓; GWP ~1.0–1.2 t CO_2_ e/t	Higher melting T; offset by durability and reuse	Higher E; lightweighting compensated cost	+10% cullet → ≈ 3% ↓ energy; GWP 1.0–1.2 t CO_2_ e t^−1^; regional loops cut emissions by ≈ 25%
Primary application sectors	Food and beverages; sauces; jars	Parenterals; diagnostics; hot-fill	Premium beverages; cosmetics; refillable containers	All sectors; deposit-return loops; amber preferred for light-sensitive products

Notes: Gas permeability is effectively zero for all glass families. UV protection depends on colour (amber/green) or coatings (e.g., ALD, sol–gel). Hydrolytic class for recycled containers varies between II and I depending on cullet composition, borosilicate fraction, and treatment conditions [12,13,21,56]. For the Recycled (cullet-rich) column, Young’s modulus and flexural strength were experimentally measured on re-melted cullet glass [66,67], whereas tensile, compressive strength, and fracture toughness (KIC) are estimated by analogy with standard soda–lime glass compositions.

### 2.1. Soda–Lime Glass for Packaging Applications

(i) Composition, network roles, and forming conditions

Soda–lime silicate glass, based on a SiO_2_–Na_2_O–CaO network, remains the dominant formulation for food, beverage, and cosmetic containers [3,55,68]. Its detailed compositional ranges are reported in Table 2. Minor oxides such as MgO and Al_2_O_3_ regulate melt viscosity, mechanical stability, and chemical durability, whereas Fe_2_O_3_ and residual K_2_O primarily reflect colour chemistry and cullet-derived variability in industrial bottle streams [13,68]. The Na_2_O/CaO balance controls viscosity, hydrolytic stability, and devitrification risk during forming and cooling [3,13,34].

Typical melting behaviour and the broad working range (10^3^–10^6^ Pa·s) enable high-throughput forming routes such as press-and-blow and narrow-neck press-and-blow, ensuring mould fidelity, dimensional reproducibility, and mechanical reliability at scale [68,69].

(ii) Industrial categories and functional differentiation

Commercial soda–lime container glass is produced in three main colour-based categories, each associated with specific optical functions and packaging uses:

- Flint (colourless): Obtained from low-iron batches (Fe_2_O_3_ ≤ 0.03–0.05 wt%) and used in food, beverage, and cosmetic packaging. Premium *extra-flint* variants employ ultra-low-iron sands and enhanced refining/decolorizing to maximize clarity for luxury beverages and perfumery.
- Amber: Generated through controlled Fe–S–C chemistry and providing UV–visible attenuation up to ~450 nm, suitable for beer, nutraceuticals, and other light-sensitive products.
- Green (emerald/olive): Obtained through regulated Fe and Cr oxide additions, widely used in beverage packaging (water, wine, oils) for aesthetic appeal and partial UV filtering.

Other tonalities (e.g., cobalt blue, black opal) exist but remain niche products and are not standard categories in large-scale soda–lime container manufacturing.

(iii) Processing innovations and functional enhancements

Narrow-neck press-and-blow (NNPB) significantly increased forming efficiency and enabled lightweighting up to 30–35% in high-throughput bottle production [7,8].

Environmental improvements have largely stemmed from high-cullet feeding: each 10% cullet reduces energy demand by ≈3% and CO_2_ emissions by ≈5%, with cradle-to-cradle LCAs reporting reductions up to ≈58% at full-cullet operation [21,70].

Melting and refining rely on optimized sulfate fining and physical aids such as gas bubbling to improve homogeneity and optical quality [69]. Post-forming thermo-chemical treatments, including sulfur-based surface de-alkalization, are routinely applied to pharmaceutical containers to enhance chemical durability [56].

Strengthening through ion exchange and steam treatment improves scratch resistance and hardness, limiting microcrack initiation [71].

Digitalization is increasingly integrated into production lines: AI-assisted inspection and predictive control support defect detection and stabilize forming conditions, improving process reliability and operator safety [72,73].

(iv) Data-driven evolution, microstructure, and limits

Recent industrial datasets and plant-scale studies indicate incremental refinements in soda–lime compositions aimed at improving durability, energy efficiency, and reuse potential. Large-scale analyses [57,68] document modest compositional shifts—particularly reduced Na_2_O in modern flint and amber formulations—and bounded adjustments in Al_2_O_3_ and MgO within established industrial windows. Fine control of Fe_2_O_3_ (<0.05 wt%) and the Fe^3+^/Fe^2+^ ratio supports colour neutrality for high-clarity flint variants used in premium packaging [58,59].

Despite these refinements, soda–lime glass retains intrinsic limits in hydrolytic performance and thermal-shock resistance under demanding conditions.

(v) Packaging applications and suitability

Soda–lime glass remains the standard material for food, beverage, and cosmetic packaging owing to its balanced optical performance, processability, and cost efficiency. Its composition supports a wide viscosity window for high-throughput forming, enabling thin-wall containers with high dimensional reproducibility. The chemical durability associated with its II–III hydrolytic class is suitable for acidic and neutral food products, sauces, beverages, and personal-care formulations, while amber and green variants extend applicability to light-sensitive products by providing intrinsic UV–visible attenuation (Table 2).

Mechanical properties—including high compressive strength, stable hardness, and rigidity—support filling, capping, transportation, and returnable systems, with failure rates primarily governed by surface flaws rather than bulk strength. Lightweighting strategies enabled by NNPB maintain required top-load performance while reducing raw-material consumption and CO_2_ emissions. Surface de-alkalization treatments extend suitability to selected pharmaceutical preparations requiring enhanced chemical stability.

Limitations persist in thermal-shock tolerance, which restricts sudden temperature gradients, and in hydrolytic resistance compared with Type I borosilicates for parenteral packaging. Nevertheless, in food, beverage, and cosmetics, soda–lime compositions define the current industrial baseline in terms of sustainability, recyclability, and functional performance.

This baseline frames the transition to borosilicate systems, which offer superior hydrolytic and thermal performance for high-stability packaging.

### 2.2. Borosilicate Glass for Pharmaceutical and High-Stability Packaging

(i) Composition, network roles, and forming conditions

Borosilicate glasses incorporate B_2_O_3_ as a second network former within typical ranges of SiO_2_–B_2_O_3_–Al_2_O_3_–alkali oxides, generating a highly polymerized silicate–borate network characterized by reduced ion mobility and superior hydrolytic resistance [6]. As alkali content increases, trigonal [BO_3_] units convert into tetrahedral [BO_4_]^−^ groups, enhancing connectivity and lowering non-bridging oxygen content [74]. This structural configuration underpins the exceptional thermal and chemical durability that makes borosilicate the reference material for parenteral containers, laboratory ware, and heat-resistant consumer packaging [3,52,54].

Primary pharmaceutical packaging is produced by tubing conversion, the industrial route for Type I borosilicate vials, ampoules, cartridges, and prefillable syringes, where Class-I hydrolytic resistance and tight dimensional control are mandatory [6]. The low thermal-expansion coefficient permits localized flame-working and precise annealing with limited stress accumulation, supporting stringent tolerances required under USP <660> [75] for stopper fit, machinability, and autoclave integrity.

Borosilicate melting and working require higher temperatures than soda–lime (~1650–1700 °C). Viscosity profiles are tuned for tubing conversion and localized flame-operations (cutting, tip-off, finish forming), ensuring that the glass remains sufficiently fluid for shaping while maintaining dimensional stability. Post-forming annealing relieves residual stresses, and alkali-depletion treatments improve inner-wall durability and minimize ion release during sterilization and storage [52,56]. In-line optical inspection may be complemented by high-resolution surface analysis techniques such as XPS, AFM, or ToF-SIMS to monitor surface cleanliness and alkali migration after flame-working [62]. These measurements feed into the control of flame-working, annealing, and de-alkalization windows to maintain both dimensional tolerances and Class-I hydrolytic performance.

(ii) Industrial categories and functional differentiation

A practical classification distinguishes three functional borosilicate families, with partial overlap across applications:

- Type I borosilicate—Used for primary pharmaceutical packaging complying with Hydrolytic Class I under USP <660> and ISO 4802 [75,76,77]. These compositions combine high silica, moderate boron, and very low alkali content to minimize ion exchange and pH shifts in injectables while ensuring low thermal expansion and high surface durability. They are employed for vials, prefillable syringes, ampoules, and cartridges [3,6,52].
- Technical borosilicate (e.g., Pyrex^®^, Duran^®^)—Glasses with very high silica and higher alkali levels than Type I, optimized for thermal-shock resistance, transparency, and durability over repeated washing or sterilization. They are not designed for extreme hydrolytic stability but are widely used in laboratory ware, bakeware, reagent bottles, and optical or photonic components [3].
- Alkali-free borosilicate—Compositions with high silica and negligible alkali oxides, replaced by alkaline-earth modifiers to suppress ionic mobility. Their low permittivity and loss tangent enable hermetic and dielectric packaging in microelectronics, RF systems, and optoelectronic devices, including substrates, optical windows, cover glasses, and interposers [46,48].

(iii) Processing innovations and functional enhancements

**Figure 6:**
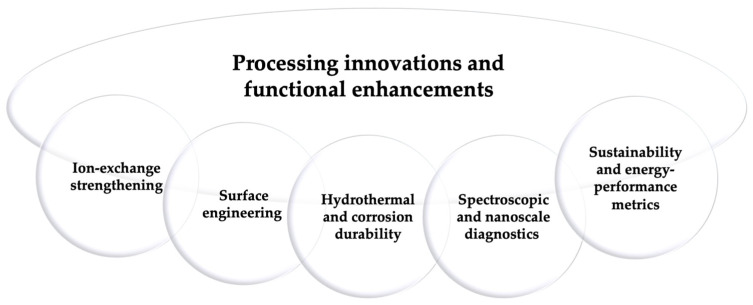
Schematic structure of the “Processing innovations and functional enhancements” block. The diagram summarizes the five thematic areas discussed in Section 2.2 (iii): ion-exchange strengthening, surface engineering, hydrothermal and corrosion durability, spectroscopic and nanoscale diagnostics, and sustainability or energy-performance metrics.

Processing developments in borosilicate glass increasingly target mechanical robustness, surface integrity, and hydrothermal durability in pharmaceutical and diagnostic packaging. These improvements are particularly relevant for vials, cartridges, cuvettes, and microfluidic components used in sample storage, transport, and in vitro diagnostic workflows. As outlined in Figure 6, this block is structured into five areas—ion-exchange strengthening, surface engineering, hydrothermal and corrosion durability, spectroscopic and nanoscale diagnostics, and sustainability or energy-performance metrics—each reflecting an active research and industrial pathway for performance optimization.

- Ion-exchange strengthening

Ion-exchange strengthening replaces near-surface Na^+^ with K^+^ through molten KNO_3_ treatment, generating a 20–50 µm compressive layer while preserving transparency and hydrolytic stability [17]. Mechanical benefits are verified through burst-pressure tests: untreated tubing vials withstand ≈36–37 bar, whereas strengthened vials exceed the 60 bar limit of standard testers, indicating at least a ≥60 bar strength [78]. The improvement follows classical fracture mechanics, as surface compression lowers the effective stress intensity at flaws and delays crack initiation [54]. In packaging practice, this reinforcement increases safety margins during fill–finish operations and supports high-pressure or concentrated drug products.

- Surface engineering

Surface engineering improves chemical stability and reduces particle shedding or drug adsorption without altering the bulk glass. Two technologies dominate:

– Atomic Layer Deposition (ALD) produces nanometric Al_2_O_3_/SiO_2_ films with excellent uniformity, sealing microdefects and reducing protein adsorption while improving abrasion resistance [48].
– Sol–gel nanocoatings allow tunable wettability and smoother inner surfaces, supporting drug recovery and mitigating residue formation.

These ultrathin coatings stabilize vial–formulation interactions, limit particulate generation during filling or transport, and complement ion-exchange strengthening in modern borosilicate packaging [62].

- Hydrothermal and corrosion durability

Borosilicate glass maintains high chemical stability under hot aqueous conditions typical of sterilization and pharmaceutical storage. Dissolution depths remain below ≈150 nm after 24 h at pH ≈ 2.4 and 70 °C [6,54], preserving dimensional accuracy and limiting leachables. Compositionally, reduced alkali and partial substitution with MgO or ZnO suppresses ion mobility across pH ≈ 2–10, improving multi-use scenarios. Borosilicates also sustain thermal gradients of ≈200–250 °C, supporting autoclaving, hot-fill, and rapid line transitions with minimal breakage.

- Spectroscopic and nanoscale diagnostics

Advanced diagnostics verify surface chemistry after forming, strengthening, and sterilization.

XPS detects alkali depletion and contamination; AFM quantifies nanoscale roughness; FTIR identifies network rearrangements; ToF-SIMS maps depth-resolved ion migration [62].

These tools support fine control of flame-working, ion exchange, annealing, and coatings, improving lot-to-lot reproducibility and compliance with GMP and traceability frameworks such as the EU PPWR [79].

- Sustainability and energy-performance metrics

Borosilicate furnaces operate at ≈1650–1700 °C and accept limited cullet fractions (≤40 wt%) to maintain hydrolytic reliability, resulting in higher energy demand than soda–lime production. Emissions reductions of ≈10–20% are achieved through oxy-fuel and hybrid-electric melting, while laser-sorting systems enable dedicated closed-loop recovery of borosilicate cullet [6,54]. Ongoing work explores lower-carbon boron sources and hybrid borosilicate matrices for waste vitrification and dielectric applications [26,80].

(iv) Microstructural refinements, composition limits, and performance boundaries

Performance boundaries in Type I borosilicate are governed by bulk composition, surface chemistry, and high-temperature processing. Even when meeting Hydrolytic Class I specifications, hot-end forming and annealing can redistribute alkalis, producing submicrometric gradients that influence reactivity. Pharmaceutical–vial studies confirm these effects, motivating post-forming stabilization such as de-alkalinization rinses or thin SiO_2_/PICVD coatings [52].

Processing adds further constraints. High melting and working temperatures (≈1650–1700 °C) and B_2_O_3_ volatilization narrow the forming window, requiring controlled flame-working and annealing. Ion-exchange strengthening provides a 20–50 µm compressive layer that improves fracture resistance, but depth and stress profiles must remain balanced to prevent subsurface tensile zones. In-line and post-forming diagnostics—XPS, ToF-SIMS—are increasingly used to validate alkali distribution and stress gradients.

Overall, bulk composition sets the hydrolytic and thermal baseline, while surface chemistry and process history define the practical performance envelope for Type I borosilicate in pharmaceutical service. Broader compositional routes for alumino-borosilicate systems targeting low coefficient of thermal expansion (CTE) and electronic or functional packaging—via BaO/SrO tuning or rare-earth co-doping [46,74,81] are covered in Section 2.5.

The next sections extend this multiscale framework to aluminosilicate and fused silica.

(v) Packaging applications and suitability

Type I borosilicate remains the reference material for parenteral packaging, where hydrolytic stability, dimensional precision, and thermal robustness ensure container integrity through sterilization, fill–finish operations, and long-term storage. Its low alkali mobility prevents pH drift and minimizes extractables, supporting sensitive formulations including biologics, mRNA systems, and high-potency injectables. These properties underpin its use in vials, ampoules, cartridges, and prefillable syringes under USP <660> and ISO 4802 compliance [75,76,77].

In diagnostic and analytical packaging, borosilicate provides optical clarity, chemical inertness, and mechanical reliability for microvials, cuvettes, reaction chambers, and microfluidic cartridges. The stability of its surface chemistry after flame-working and sterilization reduces adsorption of proteins or nucleic acids and limits particle generation during sample handling, enabling reproducible quantitative assays.

Beyond pharmaceutical and diagnostic uses, alkali-free and technical borosilicates support functional packaging in electronics and photonics, where dielectric stability, low CTE, and hermeticity are critical. These compositions are used as substrates, cover glasses, sealants, and optical windows in MEMS, RF modules, LEDs, and integrated sensing devices, benefiting from controlled thermal expansion and suppressed ionic migration.

Across these domains, suitability is governed by a balance of composition, forming history, surface condition, and microstructural stability. Their combined effect defines the operational envelope in which borosilicate glass ensures mechanical reliability, chemical neutrality, and regulatory-compatible performance across a wide variety of packaging architectures.

### 2.3. Aluminosilicate Glass

Aluminosilicate glasses are not used in mainstream pharmaceutical or food packaging, but selected low-alkali formulations have entered niche applications such as high-strength vials and diagnostic cartridges. This subsection reviews their compositional basis, processing constraints, and hydrolytic/performance boundaries under packaging-relevant conditions.

(i) Composition, network roles, and forming conditions

These glasses typically contain 5–15 wt% Al_2_O_3_ in a silicate network modified by alkali (Na_2_O/K_2_O) or alkaline-earth (CaO/MgO) oxides. Al substitutes for Si in tetrahedral coordination, forming [AlO_4_]^−^ units charge-balanced by modifiers, which increase network connectivity. This raises polymerization and reduces non-bridging oxygens, resulting in higher rigidity and improved durability under neutral to mildly acidic conditions [53].

A workable balance between viscosity and hydrolytic durability depends on the Al_2_O_3_/alkali ratio, especially in low-alkali grades developed for high-strength vials and diagnostic substrates.

(ii) Industrial categories and functional differentiation

Aluminosilicates exist as fully amorphous glasses and partially crystallized glass–ceramics. Among amorphous compositions, only a narrow subset is relevant to packaging; most are used in technical, optical, or electronic fields. Glass–ceramics such as LAS offer near-zero thermal expansion but are not used in food or pharmaceutical packaging [82].

Four main sub-classes of amorphous aluminosilicates are identified based on modifier type and concentration. Only the first category is used in pharmaceutical or diagnostic packaging; the others serve mainly structural or electronic sealing applications.

- Alkali-bearing aluminosilicates—ion-exchangeable glasses combining high rigidity with the ability to develop strong compressive layers. They are used in chemically strengthened vials and cartridges, offering lower breakage, higher dimensional robustness, and reduced extractables versus Type I borosilicate [3,6]. This is the only aluminosilicate class currently adopted in commercial primary packaging.
- Alkaline-earth aluminosilicates—used in displays, optical sealing, and functional/electronic packaging requiring low CTE; not used in direct-contact pharma packaging [60].
- Low-alkali/alkali-free aluminosilicates—for displays and multilayer sealing requiring suppressed alkali mobility; not used in pharmaceutical primary contact [83,84,85].
- High-alumina aluminosilicates—used in abrasion-resistant optical covers and high-temperature insulators; unsuitable for direct-contact packaging due to high softening temperature and limited ion exchange.

(iii) Processing innovations and functional enhancements

For aluminosilicate glasses relevant to packaging, process developments concentrate on three aspects: ion-exchange strengthening, pre-exchange thermal conditioning, and sustainability or energy-performance metrics. These routes are applied almost exclusively to alkali-bearing compositions used for high-strength vials and diagnostic substrates, where fracture resistance, reliability on fill–finish lines, and compatibility with sterilization cycles are critical.

- Ion-exchange strengthening

Alkali aluminosilicates respond efficiently to Na^+^→K^+^ exchange in molten KNO_3_ (≈400–430 °C), producing a 20–70 μm compressive layer that increases crack resistance. In vial geometries, strengthened tubing shows delayed flaw activation and higher burst strength than untreated samples [86].

- Thermal history and pre-densification

Pre-exchange thermal conditioning—via pre-densification or sub-Tg annealing—increases peak compressive stress and stabilizes the stress profile, improving vial reliability during fill–finish and high-speed transport [87].

- Sustainability and energy-performance metrics

Alkali aluminosilicates require high melting temperatures (≈1650–1750 °C) and strict purity levels for packaging and diagnostic applications, resulting in elevated furnace energy demand. Partial mitigation is achieved through oxy-fuel and hybrid-electric melting, which reduces CO_2_ emissions by >20% per ton of glass [3]. Recycling feasibility is higher in electronics and engineered-substrate streams, where provenance control and mono-material sorting are already established [6].

(iv) Microstructural refinements, composition limits, and performance boundaries

Advances in packaging-grade aluminosilicate glasses have clarified how bulk composition, network architecture, and surface processes jointly constrain performance in parenteral vials and cartridges. In alkali-bearing aluminosilicates, three relationships are particularly relevant.

- Composition and network effects

Increasing Al_2_O_3_ content promotes [AlO_4_]^−^ tetrahedra, reduces non-bridging oxygens, and improves rigidity and hydrolytic resistance. However, alumina levels above ≈18–20 wt% raise melt viscosity and softening temperature, narrowing the forming window and complicating dimensional control in tube conversion. This trade-off defines a practical upper composition limit for industrial pharmaceutical applications [53,88].

- Surface relaxation and crack initiation

Flame-based conversion of aluminosilicate tubing can induce alkali depletion and nanoscale surface relaxation, generating residual stress gradients that reduce flaw tolerance under thermal cycling or impact. Controlled annealing or surface re-equilibration is therefore required before ion exchange or direct-contact use [52,88].

- Ion-exchange and stress depth limitations

Na^+^→K^+^ ion exchange improves fracture resistance through surface compression, but the compressive layer is constrained by alkali content and pre-exchange thermal history. Excessive penetration depth (>70 μm) or unbalanced stress fields may trigger spontaneous failure during finishing or pressure testing, requiring tight control of process temperature and exchange duration [87].

Hydrolytic resistance of ion-exchanged aluminosilicate packaging glass complies with ISO 719 Class S1 [89], supporting direct-contact use in injectable and diagnostic formats that demand very low extractables [6,52,54].

Taken together, these relationships show that aluminosilicate packaging glass can outperform conventional borosilicate in crack resistance and dimensional stability, but its feasibility is bound by forming viscosity, surface relaxation kinetics, and ion-exchange process limits, which guide both current adoption and future developments in chemically strengthened container glass.

(v) Packaging applications and suitability

In the current packaging landscape, alkali-bearing aluminosilicate glasses occupy a narrow but clearly defined niche in pharmaceutical and diagnostic applications. Compared with soda–lime, they offer higher elastic modulus and improved flaw tolerance, making them suitable for high-strength vials, cartridges, and selected syringe barrels operating on fast filling lines. Relative to Type I borosilicate, they can provide superior crack resistance and dimensional stability, although their industrial adoption is limited by high melting temperatures, stricter batch purity, and reduced recycling flexibility (Table 2). For these reasons, aluminosilicate packaging glass is used where enhanced mechanical reliability justifies specialized processing, complementing rather than replacing borosilicate in high-stability parenteral containers.

Beyond pharmaceutical and diagnostic use, low-alkali and alkali-free aluminosilicates also contribute to functional and electronic packaging, serving as substrates, cover glasses, interposers, and protective or sealing layers. Their high stiffness, tailored thermal expansion, and dielectric stability (Table 3) enable reliable encapsulation of microelectronic, photonic, and sensing components. These electronic and functional packaging roles are discussed in the subsequent sections dedicated to fused silica and advanced functional glass families.

### 2.4. Recycled and Cullet-Rich Glass

Recycled glass used as cullet is the core enabler of circular manufacturing in the container-glass sector. Although it does not form a distinct compositional family, its integration into industrial batches modifies melt chemistry, furnace efficiency, process emissions, and regulatory performance.

(i) Composition, network roles, and forming conditions

In recycled and cullet-rich soda–lime glasses, cullet acts as a chemically compatible batch component replacing up to ≈80 wt% of virgin raw materials without altering network connectivity, provided feedstock purity and colour sorting are maintained [13]. Higher cullet levels reduce energy use and virgin-batch demand, while property retention depends on sorting quality and furnace conditions [7,10,13]. Because its oxide composition (SiO_2_, Na_2_O, CaO) matches that of the batch and cullet is already amorphous, incorporation lowers melting enthalpy, stabilizes furnace chemistry, and enables large-scale circularity in food and beverage packaging.

Borosilicate and aluminosilicate glasses require stricter compositional and purity control and are therefore recycled in closed or semi-closed loops with ≤30–40 wt% cullet, particularly in pharmaceutical, diagnostic, and technical applications. Functional and electronic packaging glasses—alkali-free borosilicates and high-alumina aluminosilicates—fall outside mainstream recycling and are recovered through high-purity take-back streams due to their specialized compositions and low production volumes.

In Europe, soda–lime container glass exceeds 50% cullet on average, reaching ≈95% in green bottles owing to chromophore tolerance [8,10,70]. Each additional 10 wt% cullet lowers melting energy demand by ≈3% and CO_2_ emissions by ≈5%, reinforcing its decarbonization role [14,15,90].

Cullet-rich batches retain viscosity and thermal stability compatible with high-throughput forming (press-and-blow, narrow-neck press-and-blow). Lower melting temperatures and more stable redox equilibria limit alkali volatilization and fining loads, improving mould filling and wall-thickness uniformity at industrial scale.

(ii) Industrial categories and cullet-dependent applications

The share of cullet incorporated in container glass depends on colour-specific optical and functional requirements.

- Green glass accommodates up to ≈95 wt% cullet because Fe–Cr chromophores tolerate mixed-colour feedstock, supporting beer and wine packaging.
- Amber glass typically includes 60–80 wt% cullet; Fe–S–C chromophores provide intrinsic UV shielding for light-sensitive beverages.
- Flint and extra-flint glass are generally limited to 30–50 wt% cullet since very low Fe_2_O_3_ levels are required to preserve brightness and colour uniformity [13]; used for premium transparent containers.
- Pharmaceutical and diagnostic glass (borosilicates and high-alumina alkali-free compositions) accepts lower cullet fractions or uses dedicated take-back systems due to stringent durability and clarity requirements.

In countries with advanced sorting (Switzerland, Germany, Sweden), green-glass remelting rates exceed 90%, supported by well-controlled cullet streams [8,10].

(iii) Technological and environmental advances

Recent innovations in container-glass melting have delivered measurable efficiency and emissions gains over conventional air–fuel furnaces. Oxy-fuel and hybrid-electric systems reduce fuel demand and thermal losses by eliminating nitrogen ballast, lowering fossil-fuel use and particulate emissions [21]. When combined with cullet-rich batches, they enable ~20% CO_2_ reductions at industrial scale [14,21], and batch pre-heating or heat-recovery systems further decrease energy load and flue-gas losses [10].

These gains exceed those achievable in older regenerative air–fuel furnaces, where nitrogen in combustion air increases heat loss and flue-gas volume.

- *Quality dependency.*

Substitutability modelling shows that only ≈83% of recycled cullet is effectively replaceable once colour contamination and impurities are considered, reducing environmental credit by ~13–23% relative to an ideal 1:1 assumption [12].

- *System-level corroboration.*

LCAs for beverage packaging confirm that high-cullet operation (~90 wt%) remains environmentally favourable for transport distances up to ≈250 km within regional loops, supporting coordinated technological (high-cullet melting) and environmental (short-haul closed-loop) strategies [65].

(iv) Life-cycle and policy framework

Life-cycle assessments consistently show that high-cullet operation markedly improves the environmental performance of glass packaging, particularly when supported by efficient logistics and regional closed-loop recovery [65]. Benefits are amplified when high-cullet content is combined with lightweight designs and short transport distances, as demonstrated in comparative LCA studies for hollow glass containers [91], outperforming single-lever strategies across production and use phases. The EU Packaging and Packaging Waste Regulation (PPWR) requires all packaging to be recyclable “in an economically viable way” by 2030, introduces reuse targets for beverage containers (10% by 2030, rising to 40% by 2040), and maintains glass-recycling targets of 70% (2025) and 75% (2030) [79].

Industry initiatives coordinated by FEVE support high-cullet batches, bottle-to-bottle closed loops, and traceable supply chains aligned with SDG 12 and Green Deal objectives [70]. Independent analyses confirm that glass retains its circular advantages only when these combined measures operate across the value chain [92].

Overall, cullet-rich formulations, integrated reuse schemes, and policy-driven recyclability criteria reinforce the role of glass as a permanently recyclable and environmentally compatible packaging material.

(v) Packaging applications and suitability

Recycled and cullet-rich soda–lime glass is widely used in food, beverage, cosmetic, and household packaging, where it maintains mechanical strength, chemical inertness, and optical stability across multiple remelts. High-cullet bottles (≈60–90 wt%) show clear environmental advantages in LCAs for water, wine, and craft-beer packaging, with reduced GWP and energy demand, especially when transport remains within regional loops [14,65,93].

Green and amber bottles tolerate the highest cullet fractions and dominate beer and wine, while flint and extra-flint remain suitable for premium food and cosmetic containers when colour purity is controlled [18,94]. Substitutability models indicate that ≈83% of cullet can replace a virgin batch with proper colour sorting [12].

Non-container outlets—bricks, tiles, architectural and design components—absorb mixed or lower-quality cullet unsuitable for bottle-to-bottle cycles [66,95,96].

Overall, cullet-rich glass remains suitable for high-volume packaging, reuse schemes, and stable secondary markets, supported by reduced melting energy (–2.68 GJ t^−1^) and strong circularity performance.

### 2.5. Functional, Electronic, and Specialized Packaging Glasses

Glass packaging has expanded from passive containment to a structural and functional element within integrated devices. Its combination of transparency, chemical stability, electrical insulation, and tunable thermal expansion—critical for matching Si, GaN, or Al_2_O_3_—enables hermetic sealing, dielectric insulation, optical interfacing, and substrate-level support in miniaturized assemblies [40,41,43,44].

**Figure 7:**
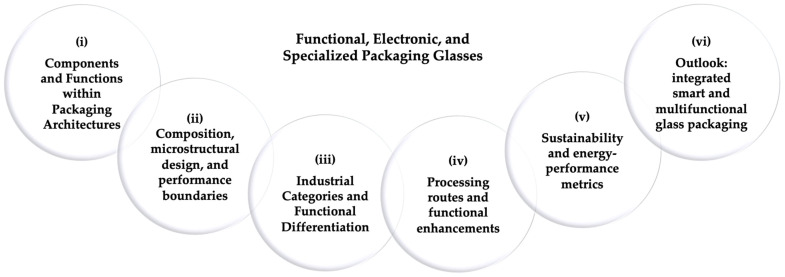
Structured overview of the six analytical layers used to characterize functional, electronic, and specialized packaging glasses. The sequence progresses from component-level roles (Section i) to compositional and network design (Section ii), industrial classification (Section iii), processing innovations (Section iv), and microstructural performance boundaries (Section v), concluding with emerging directions in smart and multifunctional packaging architectures (Section vi).

As packaging has evolved from macroscopic housings to micro- and nanoscale architectures, glass has become one of the few materials capable of simultaneously fulfilling structural, dielectric, and optical requirements. Within such architectures, glass operates as an integral part of the device, forming capping lids, sealing frames, optical windows, or interposers with through-glass vias through anodic bonding, glass-frit bonding, or wafer-level vacuum sealing [40,41,43,44]. In these roles it defines interfaces and encapsulating layers that guarantee environmental protection, dimensional stability, electrical isolation, and controlled light transmission or thermal management. These functions underpin the reliability of MEMS, infrared sensors, integrated photonic components, and multilayer electronic or RF modules [45,46,47,48]. This historical shift from sealing medium to system-level material provides the basis for the framework developed in this section. The analysis proceeds from component roles to compositional design, industrial classification, processing routes, and microstructural performance, concluding with emerging multifunctional glass packaging architectures (Figure 7).

(i) Glass Components and Functions within Packaging Architectures

**Figure 8:**
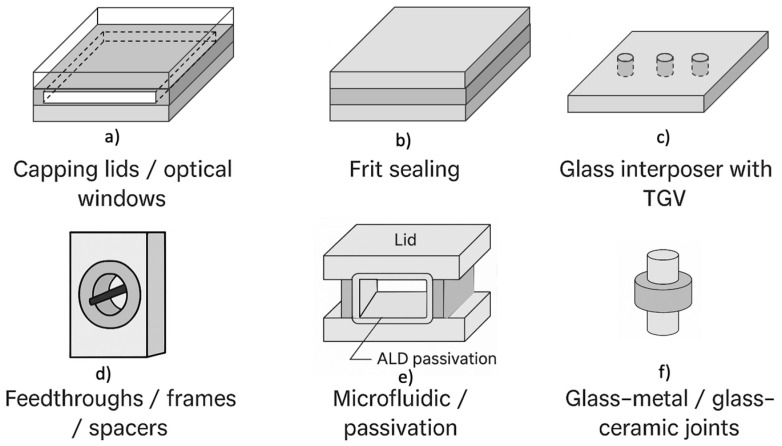
Schematic representation of the six principal glass components used in functional and electronic packaging architectures: (**a**) capping lids and optical/IR windows; (**b**) sealing frit layers; (**c**) glass interposers with through-glass vias (TGVs); (**d**) glass feedthroughs, frames, and spacers; (**e**) microfluidic and passivation layers; (**f**) glass–metal and glass–ceramic joints for harsh environments.

In contemporary encapsulation systems, glass functions as an integrated structural and dielectric element that defines interfaces, optical paths, and barrier layers at the micro- and mesoscale. Its configurations fall into six functional sets—illustrated in Figure 8—which often coexist within the same device. Table 1 provides the corresponding functional classification, summarizing structural and dielectric roles, sealing modes, and typical material families.

- Capping lids and optical/IR windows.

Glass wokafers or frames act as hermetic covers and optical windows, providing controlled vacuum or gas environments for MEMS and sensors, mechanical alignment, and visible/IR transmission [40,43]. Material selection—low-CTE borosilicates, IR-transparent glasses, or low-expansion LAS glass–ceramics—depends on thermal matching, optical losses, and chemical stability [82,97]. Hermetic closure is achieved through anodic, frit, or laser bonding. Pb-free frits such as BaO–B_2_O_3_–ZnO and Bi–B–Zn systems enable low-temperature sealing and stable peripheral frames for wafer-level encapsulation [41,98,99]. As shown in Figure 8a, the glass lid bonds to a sealing frame enclosing a vacuum or gas-filled cavity while allowing optical or IR transmission.

- Sealing media and hermetic interfaces.

Lead-free frits based on Bi–B–Zn or Ba–Zn–B_2_O_3_ and glass-to-silicon or glass-to-ceramic joints form dense, low-porosity, high-wettability interfaces that provide long-term hermeticity [98,99,100,101]. Anodic bonding offers thin, electrically insulating interfaces with controlled residual stress [40]. These sealing elements define the package volume, thermal-cycling resistance, and electrical isolation. As shown in Figure 8b, the hermetic layer forms a continuous joint between glass and substrate, ensuring mechanical integrity and gas impermeability.

- Dielectric substrates and interposers (with TGVs).

Glass substrates with through-glass vias (TGVs) provide vertical feedthroughs that connect sealed MEMS or sensor cavities to external circuitry while preserving planarity and hermetic boundaries [44,50]. The glass plate acts simultaneously as mechanical support and a low-loss dielectric medium, with metallized vias ensuring electrical continuity without degrading sealing performance.

Beyond hermetic MEMS modules, TGV-equipped substrates function as dielectric interposers for 2.5D/3D integration. Mixed alkaline-earth aluminoborosilicate glasses offer controlled CTE, dimensional stability, and low dielectric loss, supporting high-density redistribution layers and Cu-filled TGVs [45,46]. Reliability depends on Cu–glass thermomechanical interactions, via-filling uniformity, and multilayer stress evolution, with failure modes such as radial cracking, Cu protrusion, and interfacial delamination [51].

As illustrated in Figure 8c, metallized TGVs traverse the glass thickness, providing vertical interconnection while maintaining structural and dielectric functions.

- Feedthroughs, frames, and structural spacers.

In wafer- and device-level packaging, glass also acts as an internal structural element. Glass frames form the lateral walls of cavities in MEMS inertial sensors, pressure sensors, microfluidic chips, and IR detectors, ensuring alignment between lid and device wafer. Spacers maintain stand-off distance in stacked wafers, preserving planarity and preventing die deformation in resonant and cavity-based devices.

Metallized vias or holes within these frames create insulated feedthroughs that route electrical or fluidic connections across the package boundary while preserving dielectric isolation. They appear in pressure sensors with peripheral routing, sealed-channel microfluidics, and MEMS modules with perimeter feedthroughs for ASICs. Glass in these roles provides rigidity, dimensional tolerance, breakdown strength, ageing resistance, and compatibility with anodic or frit bonding. Performance depends on CTE matching, metallization adhesion, and bonding-interface reliability [25,26]. As shown in Figure 8d, a glass frame can integrate a metallized feedthrough while acting as the spacer defining cavity height.

- Microfluidic and passivation layers.

In microfluidic and lab-on-chip devices, glass forms the packaging architecture itself: etched microchannels and sealed cavities act as inert, sterilizable micro-containers for fluids, reagents, and biological samples, providing chemical compatibility, optical access, and dimensional stability [24,102]. Glass simultaneously confines the fluid, maintains flow-path geometry, supplies mechanical rigidity, and provides a transparent window for optical interrogation.

As shown in Figure 8e, bonded lids seal etched microchannels to form enclosed cavities, while ALD passivation stabilizes chemical, dielectric, and moisture-barrier performance.

In electronic, photonic, and biomedical devices, thin glass passivation layers or glass/oxide stacks act as dielectric barriers that suppress ion migration, limit leaching, and protect components from moisture and ageing. Nanoscale ALD films of SiO_2_ or Al_2_O_3_, originally developed for pharmaceutical primary packaging [20], are increasingly used in microsensors and microfluidic platforms to enhance long-term stability and barrier performancece [27].

Across these systems, glass microstructures and passivation layers define closed environments and ensure optical, chemical, mechanical, and dielectric integrity at the micro- and nanoscale, providing multifunctional encapsulation beyond simple containment.

- Glass–metal and glass–ceramic joints for harsh environments.

In power modules, high-temperature probes, and containment systems, glass–metal and glass–ceramic seals serve as primary packaging elements that transmit load, maintain hermeticity, and provide insulation under thermal shock or corrosive exposure [26,28]. Typical implementations include compression and matched seals where glass or glass–ceramic rings bond metallic pins or housings, ensuring mechanical restraint, dielectric isolation, and long-term leak tightness [26,103]. Material selection—borosilicate or aluminosilicate glasses with ZrO_2_/TiO_2_ or LAS glass–ceramics—is governed by CTE matching, bonding-temperature viscosity, and resistance to devitrification and corrosion [82]. As illustrated in Figure 8f, the glass joint forms an insulating barrier between metallic components while accommodating thermal stresses and preserving hermeticity under severe mechanical, thermal, and chemical loads.

In all these configurations (Figure 8), glass plays a dual role: structural—by defining geometry, load paths, and tolerances—and functional, by providing optical, dielectric, or barrier performance. Composition and processing are chosen not only for formability but to meet system-level requirements such as thermal compatibility with substrates, RF-loss minimization, IR transmittance, device temperature limits, and reliability under HTOL (High-Temperature Operating Life) or thermal cycling. This perspective frames the following subsections, which analyze how specific glass families and processes realize these functions within defined microstructural, energetic, and sustainability constraints.

(ii) Composition, microstructural design, and performance boundaries

Functional and electronic packaging glasses derive from a restricted set of high-purity borosilicate, fused-silica, aluminosilicate, and hybrid formulations engineered for hermetic sealing, dielectric insulation, optical transmission, and structural stability. These materials enable packaging functions in microsystems, sensors, microfluidic platforms, optical modules, and high-reliability electronic assemblies, where glass must provide chemical durability, controlled thermal expansion, optical access, and compatibility with bonding or metallization. Table 3 reports representative compositions and property ranges for the glass families most widely used in these scenarios.

The analysis links bulk composition and network roles to the microstructural limits and performance windows that define their applicability in advanced packaging.

- Glass families, compositions, and property ranges

Composition determines network connectivity, polarizability, viscosity fields, and the forming conditions that enable reproducible interfaces and mechanically stable joints.

- High-purity borosilicate glasses

Low-alkali borosilicates (≈77–80 wt% SiO_2_, 9–13 wt% B_2_O_3_, ≤6 wt% alkalis, 2–6 wt% Al_2_O_3_) offer moderate thermal expansion (CTE 3.2–3.4 × 10^−6^ K^−1^), low dielectric loss, and high optical transmittance, supporting their use in capping lids, optical/IR windows, and microfluidic substrates (Figure 8a,e). Their network comprises interconnected SiO_4_ and BO_4_ units with limited non-bridging oxygens, while alkali or alkaline-earth modifiers tune viscosity and bonding behaviour. These glasses are widely used in wafer-level MEMS encapsulation and diagnostic platforms due to their stability under anodic and frit bonding [17,40,43,104].

- Fused silica

Fused silica (>99.8 wt% SiO_2_) is an ultra-high-connectivity network glass with minimal modifiers, giving extremely low CTE (≈0.5 × 10^−6^ K^−1^), broad optical transparency, and high thermal-shock resistance. Its high viscosity requires laser- or plasma-assisted processing, but it offers unmatched dimensional stability for precision lids and windows in high-accuracy sensing and microfluidic systems (Figure 8a,e). It is the standard material for optical diagnostics requiring UV–IR access and minimal deformation [24,105,106].

- Boro-aluminosilicate glasses

Boro-aluminosilicate compositions with BaO and ZnO (≈70 wt% SiO_2_, ≈12 wt% B_2_O_3_, ≈10 wt% Al_2_O_3_, ≈5 wt% alkaline earths) offer controlled thermal expansion (CTE ≈4 × 10^−6^ K^−1^) and a stable dielectric response. Their mixed Si–B–Al network incorporates [AlO_4_]^−^ units balanced by alkaline-earth oxides, while BaO/ZnO tune viscosity and wetting at sealing temperatures. These glasses support hermetic biomedical cartridges, MEMS housings, and optoelectronic assemblies (Figure 8b–d), where dimensional stability and moderate permittivity are required [48,102].

- Low-alkali aluminosilicate glasses for TGV substrates

Aluminosilicates (≈68–70 wt% SiO_2_, 9–12 wt% Al_2_O_3_, ≤3 wt% alkalis, with MgO/BaO/ZnO modifiers) provide CTE 3.5–4.5 × 10^−6^ K^−1^, high hardness, and excellent dielectric stability, enabling their use as dielectric substrates and through-glass-via interposers (Figure 8c). Their network of SiO_4_ and [AlO_4_]^−^ units stabilizes dielectric response, while modifiers tune CTE and flow behaviour during via formation and Cu-filling. These glasses support high-density redistribution layers and reliable via–metal interfaces in 2.5D/3D microsystems and wafer-level MEMS packaging [44,45,46,51].

- Hybrid aluminosilicate–phosphate glasses

Hybrid Al–Si–P glasses (≈60 wt% SiO_2_, 10 wt% Al_2_O_3_, 10 wt% P_2_O_5_ with CaO/MgO/ZnO modifiers) combine low-temperature formability (Tg ≈ 350 °C) with strong adhesion to polymers and metals. Their mixed SiO_4_–AlO_4_–PO_4_ network provides structural flexibility and reactive sites that enable multimaterial 3D-printed microfluidic encapsulation (Figure 8e). These formulations are key for integrated lab-on-chip platforms and low-temperature microfluidic sealing [24].

- Lead-aluminosilicate and passivation-grade glasses

Lead-aluminosilicate glasses (≈20 wt% PbO, 5 wt% Al_2_O_3_, 75 wt% SiO_2_) form dense [PbO_4_] units upon annealing, increasing the bandgap and reducing mobile charge density. These properties enhance dielectric stability and moisture resistance in thin-film passivation stacks deposited onto glass substrates for sensors and photonic devices (Figure 8e). Their forming conditions rely on moderate-temperature processing and controlled oxidation environments [49].

- Borosilicate–aluminosilicate hybrid systems for harsh environments

Hybrid borosilicate–aluminosilicate compositions (≈74 wt% SiO_2_, 10 wt% B_2_O_3_, 8 wt% Al_2_O_3_, ≈8 wt% modifiers) achieve CTE ≈3.0–3.5 × 10^−6^ K^−1^ and enhanced resistance to devitrification and corrosion. Their mixed network stabilizes thermal expansion and wetting during high-temperature bonding. These glasses are used in harsh-environment joints (Figure 8f), including nuclear-adjacent sensors and long-term hermetic modules [26,28].

- Lithium-aluminosilicate (LAS) glass–ceramics

LAS glass–ceramics (Li_2_O–Al_2_O_3_–SiO_2_ with TiO_2_/ZrO_2_ nucleators) crystallize into low-expansion β-quartz solid solutions, giving near-zero CTE (0 ± 0.07 × 10^−7^ K^−1^), high rigidity, and optical transparency. These properties support their use in precision windows and glass–ceramic joints exposed to severe thermal and mechanical stress (Figure 8a,f). Their forming requires controlled nucleation and crystallization schedules to preserve transparency and dimensional accuracy [82].

- Microstructural limits and performance windows

The functional behaviour of high-purity borosilicate, aluminosilicate, hybrid, and glass–ceramic systems is constrained by microstructural limits governing processing stability, dielectric reliability, optical quality, and long-term hermeticity. These boundaries define the stability windows within which the above compositions can be used in advanced packaging. In low-alkali aluminosilicate and boro-aluminosilicate glasses for TGV substrates and high-frequency modules, alkaline-earth variations modify network polymerisation and the fraction of bridging oxygens. Increasing the Ba/Sr ratio compacts the Si–Al–O network, reduces vibrational disorder, lowers the CTE from ≈5.1 to 4.3 × 10^−6^ K^−1^, and stabilizes the relative permittivity at ε_r_ ≈ 4.5–4.6 (Xie 2024; Liu 2025) [45,46]. Rare-earth substitution (La→Gd) increases [AlO_4_] connectivity, suppresses non-bridging oxygens, and reduces dielectric loss to tan δ < 5 × 10^−4^, a lower limit for leakage-free RF and microelectronic encapsulation [46].

In Bi- and Zn-borate frits, the high polarizability of [BiO_3_]/[BiO_4_] units widens the electronic bandgap from ≈2.9 to 3.4 eV and lowers the softening temperature to ≈420 °C, enabling low-temperature sealing [49]. Above ≈80 mol% Bi_2_O_3_, phase separation and volatilisation reduce melt stability, while ZnO contents >30 mol% in Ba–Zn–B_2_O_3_ glasses promote surface crystallization and opacity [98,100]. These limits set the compositional ceiling for transparent, process-stable frits used in wafer-level bonding.

In LAS glass–ceramics for zero-expansion windows and harsh-environment supports, the crystallized fraction and crystal-size distribution define the boundary between acceptable and degraded optical performance. Nucleator levels and thermal schedules forming β-spodumene or β-quartz solid solutions at controlled volume fractions yield near-zero expansion (0 ± 0.07 × 10^−7^ K^−1^) with high transparency. Excess crystallization (>20 vol%) increases scattering and reduces transmittance, limiting LAS use in photonic and IR packaging [82].

Nanoscale ALD coatings of Al_2_O_3_ or SiO_2_ on borosilicate and aluminosilicate surfaces create dense, defect-poor barriers that suppress Na^+^ and B^3+^ migration and reduce water-vapour permeability below 10^−4^ g m^−2^ day^−1^ [20,27]. These refinements preserve optical transmittance (>90%) and prevent dielectric drift under humidity and temperature cycling, defining an upper reliability limit for hermetic micro-packages in pharmaceutical, diagnostic, and electronic devices. In aluminosilicate and LAS substrates, ion-exchange strengthening (Na^+^ ↔ K^+^) generates compressive layers >400 MPa and increases flexural strength by 2–3× without compromising dielectric or optical behaviour, provided diffusion depths remain ≤≈50 µm [25,86]. Greater depths produce steep stress gradients and residual birefringence, setting the mechanical limit for strengthened transparent components used as lids, windows, or RF carriers.

Taken together, these microstructural constraints define the practical performance envelope for functional packaging glasses:

- Relative permittivity ε_r_ ≈ 4–6 with dielectric loss tan δ < 0.005;
- Thermal expansion CTE ≈3–5 × 10^−6^ K^−1^ for matching Si, GaN, and Al_2_O_3_;
- Softening range ≈ 350–500 °C for low-energy frit or glass–ceramic sealing;
- Optical transparency > 90% in the relevant spectral window;
- Flexural strength > 400 MPa after ion exchange;
- Water-vapour transmission < 10^−4^ g m^−2^ day^−1^ with ALD barriers.

These limits complement the compositional map of the main glass families and define the quantitative boundaries within which borosilicate, aluminosilicate, hybrid, and glass–ceramic systems can operate in miniaturized, high-density, high-reliability packaging.

(iii) Industrial Categories and Functional Differentiation

Functional and electronic packaging glasses constitute specialized industrial families developed to ensure hermetic sealing, dielectric insulation, optical access, and chemical stability in microsystems and high-reliability electronic assemblies. Their differentiation reflects the dominant functional constraint of each application—thermal compatibility, optical transmission, environmental durability, or electrical insulation—rather than conventional container-glass requirements. The categories presented below correspond to the glass families in Table 3 and to the packaging configurations introduced earlier.

- Low-temperature sealing and frit glasses

These compositions enable hermetic bonding in MEMS, sensor modules, and power-electronics packages where thermal budgets are restricted. Bi_2_O_3_–B_2_O_3_–ZnO and Ba–Zn–B_2_O_3_ frit systems offer controlled softening compatible with silicon, alumina, and metal substrates, supporting wafer-level sealing and perimeter encapsulation [98,99,100]. Their processing stability and Pb-free formulations meet RoHS/REACH requirements and constitute the industrial basis for low-temperature hermetic assembly.

- Dielectric and encapsulation glasses

Used in interposers, passivation layers, and glass-to-metal or glass–ceramic sealing, these glasses ensure electrical insulation, thermal compatibility, and dimensional stability. Low-alkali aluminosilicates and alumino-borosilicates with alkaline-earth or rare-earth modifiers enable reliable bonding and stable dielectric behaviour in high-frequency or high-temperature packages [25,45,46]. Their role spans from structural support in TGV interposers to insulating barriers in harsh-environment microelectronic modules.

- Biomedical and microfluidic glasses

Borosilicate, aluminosilicate, and hybrid phosphate glasses are used in diagnostic cartridges, lab-on-chip systems, and bio-integrated sensors, where transparency, clean surfaces, and chemical inertness govern performance [24,27,102]. Their compatibility with microfabrication routes and with nanoscale ALD passivation layers enables stable microchannel architectures and long-term fluidic sealing.

- Photonic and optical-functional glasses

These glasses offer controlled refractive index, high transparency, and thermal stability for optical windows, IR detectors, and mid-IR integrated devices. LAS glass–ceramics with tailored CTE, together with transparent chalcogenide and zinc-borosilicate compositions, support optical routing, filtering, and spectroscopic sensing in mixed-wavelength photonic systems [82,97]. In functional and electronic packaging glasses, refractive index is strongly composition- and application-dependent, being governed by oxide chemistry, network modifiers, and electronic polarizability. Consequently, the discussion focuses on compositional drivers and qualitative trends rather than on single numerical values, as a unified quantitative comparison would not capture the wide heterogeneity of this glass family.

- Extreme-environment and containment glasses

Borosilicate–aluminosilicate hybrids and LAS glass–ceramics are used in high-temperature, radiation-exposed, and corrosive environments—including fuel-cell stacks, aerospace sensors, and vitrification matrices [25,26,28]. Their chemical durability, crystallization stability, and compatibility with metal housings ensure long-term hermeticity under severe thermo-mechanical and chemical loads.

Taken together, these categories show that industrial packaging glasses are differentiated primarily by function rather than by composition. Their roles span structural and hermetic interfaces, optical and dielectric elements, and microfluidic architectures, forming a continuum of packaging functions across microelectronics, photonics, biomedical systems, and extreme-environment technologies.

(iv) Processing routes and functional enhancements

Processing innovations in functional and electronic packaging glasses operate on three levers that control hermeticity, surface stability, optical quality, and long-term dielectric reliability: (i) bonding and sealing, (ii) surface or near-surface strengthening and barrier modification, and (iii) additive or hybrid micro-fabrication. Their combined action governs interface precision, encapsulation robustness, and integration with electronic, photonic, and microfluidic substrates.

- Bonding and sealing technologies

Wafer-level bonding and localized micro-sealing enable hermetic encapsulation of MEMS, sensors, optoelectronic modules, and microfluidic devices. Pb-free Bi_2_O_3_–B_2_O_3_–ZnO frits sinter at 350–450 °C, forming dense joints with leak rates below 10^−8^ mbar L s^−1^ and sub-millimetric tolerances [98,99,100] while meeting RoHS/REACH constraints and protecting temperature-sensitive components.

Diffusion bonding and anodic sealing with silicon or ceramic substrates produce interfaces with CTE mismatch < ±0.5 × 10^−6^ K^−1^ and long-term structural and dielectric stability [41,49,101]. Laser-assisted sealing and micro-jet-deposited frits further improve local control, supporting patterned sealing frames for high-density and hybrid device layouts.

Collectively, these bonding routes provide precise control of interfacial stress, hermeticity, and chemical compatibility, enabling reliable wafer-level packaging under defined atmospheric or thermal conditions.

- Surface strengthening and nanoscale modifications

Surface and near-surface treatments improve mechanical robustness, barrier performance, and dielectric stability. Ion-exchange strengthening in alkali-free aluminosilicate and LAS glasses (Na^+^ ↔ K^+^) generates compressive layers > 400 MPa and 2–3× flexural-strength gains while maintaining >90% optical transmission and ε_r_ ≈ 4.5 [82,86]. These treatments reinforce lids, interposers, and optical windows against handling, cycling, and shock.

Atomic Layer Deposition (ALD) of SiO_2_ or Al_2_O_3_ suppresses alkali migration, lowers moisture permeability by >100×, and improves resistance to chemical leaching on borosilicate and aluminosilicate surfaces [20,27]. Initially developed for pharmaceutical packaging, ALD coatings now support microfluidic stability, long-term sensor performance, and dielectric protection in photonic and microelectronic devices.

Together, these surface-engineering processes stabilize near-surface regions against mechanical, chemical, and environmental degradation, sustaining packaging performance over long service lifetimes.

- Additive and hybrid micro-manufacturing routes

Additive and hybrid fabrication methods enable geometric and architectural integration of glass in advanced packaging. Phosphate–silicate systems with Tg ≈ 350 °C allow multi-material 3D printing of glass–polymer microfluidic modules that combine electrical insulation, optical transparency, and structural integrity [24]. Low-melting Bi- and Zn-borate frits can be ink-deposited and laser-sintered to create patterned sealing paths, localized encapsulation features, and micro-interconnects [49,65], enabling complex geometries and hybrid architectures unattainable with conventional melting or planar bonding.

In this way, additive micro-fabrication expands the packaging design space, integrating optical windows, fluidic cavities, dielectric barriers, and interconnects directly into the encapsulating glass layer and supporting fully miniaturized solutions.

- Functional outcomes of combined processing routes

The combined action of bonding strategies, surface/near-surface strengthening, and hybrid or additive fabrication routes yields packaging glasses with

- Hermetic leak rates < 10^−8^ mbar L s^−1^;
- CTE-matched interfaces within ±0.5 × 10^−6^ K^−1^;
- High mechanical reinforcement (surface σc > 400 MPa; strength gains 2–3×);
- Optical retention > 90% after ion-exchange or coating;
- Enhanced barrier performance, with moisture/ion permeability reduced by ×100;
- Stable dielectric response, with ε_r_ ≈ 4.5 and low loss under thermal/humidity stress.

These performance enhancements reflect the transition from conventional melting and forming to micro-engineered processing, enabling glass to act not only as a structural material but as a multifunctional platform for advanced, miniaturized, and sustainable packaging architectures.

(v) Sustainability and energy-performance metrics

Although produced in smaller volumes than container or flat glasses, functional and electronic packaging glasses are increasingly evaluated in terms of energy and material efficiency. The transition to low-temperature, Pb-free, and recyclable formulations reduces melting energy and eliminates lead volatilization [49,98,99,100]. Bi_2_O_3_–B_2_O_3_–ZnO and Ba–Zn–B_2_O_3_ frits operate 150–200 °C below lead-borate counterparts, lowering process energy by ≈20–25% per cycle [21,90].

Hybrid-electric and oxy-fuel melting systems, already used in container glass, are now adopted for precision frits and specialty glasses. Their higher combustion efficiency and reduced NO_x_ output decrease specific CO_2_ emissions, aligning with BAT/BREF directives and industrial decarbonization targets under the Green Deal [18,66,70].

Sustainability gains also derive from batch reformulation and closed-loop recovery. Production residues and off-spec frits can be re-melted or milled as secondary cullet, benefiting from their silica–borate base and absence of alkali contamination. In specialized sectors, take-back systems are emerging for diagnostic and photonic components to recover high-purity borosilicate or aluminosilicate fragments [65,90].

At device level, the long lifetime of hermetic glass encapsulation reduces waste and replacement frequency in electronics, sensors, and biomedical cartridges. Glass passivation layers and ALD-coated borosilicates resist corrosion and ion diffusion far beyond polymeric or metallic alternatives, extending service life by factors of 5–10 [20,27]. This longevity contributes significantly to material circularity and life-cycle efficiency [18,21].

Policy frameworks now explicitly include functional and specialty glasses within Europe’s circular-material strategy. The PPWR 2025 identifies glass as a priority circular material, while the ESPR promotes recyclability, traceability, and low-carbon manufacturing in electronic components. Together, these measures extend the sustainability paradigm from mass packaging to high-performance and micro-scale glass applications, linking decarbonization targets with technological innovation.

(vi) Outlook: Integrated smart and multifunctional glass packaging

The evolution of these specialized glass families signals a shift from passive containment to multifunctional and intelligent packaging, where hermetic sealing, optical interfacing, and dielectric insulation are integrated within low-energy, recyclable, and RoHS-compliant platforms (European Parliament and Council). By combining controlled thermal expansion, low dielectric loss, and high optical transmittance, functional and electronic packaging glasses support wafer-level encapsulation of MEMS and sensors, microdiagnostic cartridges, solid-state battery housings, and IR photonic modules within a unified material framework. Their modularity and long-term stability align with European Green Deal and PPWR 2025 objectives, positioning glass as a key enabler of next-generation sustainable packaging. Future developments will leverage hybrid glass–ceramic systems, additive microfabrication, and nanoscale coatings to co-design packaging and device functions, further merging optical, electronic, and environmental performance within recyclable glass-based platforms.

### 2.6. Comparative Overview

The data summarized in Table 2 provide a quantitative baseline for conventional packaging-glass families, linking composition to key mechanical, thermal, and chemical parameters. “Type I/III Borosilicate” follows pharmacopeial definitions (USP <660>; Ph. Eur. 3.2.1), while “Aluminosilicate” denotes high-alumina soda–lime container glass, as summarized in Table 2, and should not be confused with the technical ion-exchanged aluminosilicates reported in Table 3. These values underpin assessments of reliability, processability, and environmental performance across food, cosmetic, and pharmaceutical packaging.

Table 3 extends this framework to functional and electronic packaging glasses with ultra-low CTE, high dielectric stability, and optical robustness. Here “borosilicate” and “aluminosilicate” refer to high-purity, low-alkali functional compositions designed for hermetic sealing, microfluidics, photonics, and electronic integration. Their inclusion broadens the comparison from structural packaging to multifunctional encapsulation and highlights the convergence between composition, functional performance, and circular manufacturing strategies.

**Table 3:** Functional and electronic packaging glasses: composition, properties, and applications. Comparative overview of glass systems designed for diagnostic, electronic, and extreme-containment functions. Data summarize representative studies on borosilicate, aluminosilicate, and hybrid formulations optimized for hermeticity, dielectric strength, and optical performance.

Property	Borosilicate (High-Purity, Functional)	Fused Silica	Boro-Aluminosilicate	Aluminosilicate (Ion-Exchanged/Technical)	Hybrid Aluminosilicate–Phosphate	Borosilicate–Aluminosilicate Hybrid	Low-Alkali Aluminosilicate
Reference	[17,48,60,61,104,107,108,109]	[24,60,61,105,106,107,110,111]	[41,60,61,102,112,113]	[25,60,61,86,107,110,114]	[26,61,109,111,113]	[28,41,61,107,114]	[27,60,61,109,110,111]
Main Composition (wt%)	SiO_2_ 77–80; B_2_O_3_ 9–13; Na_2_O/K_2_O 4–6; Al_2_O_3_ 2–6; CaO 1–2	SiO_2_ > 99.8	SiO_2_ ≈ 70, B_2_O_3_ 12, Al_2_O_3_ ≈ 10, BaO ≈ 3, ZnO ≈ 2	SiO_2_ 68–70, Al_2_O_3_ 9–12, Na_2_O/K_2_O ≤ 3	SiO_2_ 60, Al_2_O_3_ 10, P_2_O_5_ 10, CaO 8, MgO 6, ZnO 4	SiO_2_ 74, B_2_O_3_ 10, Al_2_O_3_ 8, Na_2_O/K_2_O 4, CaO 3	SiO_2_ 67, Al_2_O_3_ 11, MgO 7, BaO 5, ZnO 3
CTE (×10^−6^ K^−1^)	3.2–3.4 *	0.50–0.55	≈4.2	3.5–4.5 *	≈3.5 *	3.0–3.5 *	3.5–4.0
Dielectric Strength (kV mm^−1^)	20–40 *	20–25 *	16–18 *	18–22 *	15–20 *	14–16 *	≈20 *
Permittivity ε′ (1 MHz)	4.6–5.2 (lit.)	3.8–4.0 (std.)	6–8 (lit.)	6.5–11 (lit.)	12–18 (lit.)	6–8 (lit.)	6–8 (*est.*)
Loss tangent tan δ (1 MHz)	0.003–0.008 (lit.)	0.0001–0.0002 (std.)	0.001–0.004 (lit.)	0.002–0.01 (lit.)	0.005–0.015 (lit.)	0.001–0.004 (lit.)	0.001–0.004 (est.)
Hermeticity (mbar·L·s^−1^)	≤10^−8^–10^−9^ *	≤10^−9^ *	≤10^−9^ *	10^−8^ *	<10^−8^ *	10^−8^ *	10^−8^ *
Optical Transmittance (%)	>90 (400–700 nm) *	>92	88–90	88–91	85–90 *	80–85 *	>90 *
Hardness (GPa)	5.6–6.0	≈6.0 *	≈5.8	6.2–7.7 (measured; ≈620–773 HV)	≈6.2 *	≈6.0 *	≈6.8 *
Key characteristics (qual.)	Low-alkali, good CTE match to Si; stable dielectrics; low autofluorescence	Ultra-low CTE; highest optical purity; excellent radiation stability	Balanced CTE; good mechanical strength; compatible with low-T frit sealing	Chemically strengthenable; high surface hardness; impact-resistant cover glass	Low-temperature sealing; tailored CTE; good dielectric performance	Intermediate CTE; robust under thermal cycling; hermetic encapsulation	Stable permittivity; low ion migration; high dielectric reliability
Recyclability and cullet (qual.)	Not compatible with container-glass cullet; niche, small-scale recycling only	No established large-scale recycling; re-melting limited to specialty lines	Very limited recyclability; composition not accepted in soda–lime cullet loops	Very limited recyclability; composition not accepted in soda–lime cullet loops	No closed-loop routes; treated as specialty waste at end-of-life	No closed-loop routes; treated as specialty waste at end-of-life	Not accepted in mixed cullet; requires dedicated recovery to avoid contamination
Sustainability Impact	Higher melting energy than container glass; low glass mass per device mitigates impact	Very high melting energy and CO_2_ per kg; use restricted to high-value components	Energy-intensive melting; Zn/Ba oxides raise environmental and end-of-life concerns	High energy demand for melting and ion exchange; long service lifetime partly offsets footprint	High energy demand for melting and ion exchange; long service lifetime partly offsets footprint	Specialty compositions; decarbonisation relies on furnace electrification and optimized batching	Specialty compositions; decarbonisation relies on furnace electrification and optimized batching
Main Application	Microfluidic chips, biosensors, RF/microwave substrates, pharma vials	Lab-on-chip, optical diagnostic systems	Sealing for biomedical cartridges	Displays, sensors, protective windows, LED/PV modules	Micro-battery sealing, MEMS	Nuclear waste immobilization, high-temperature sensors	Transparent hermetic coatings

Notes: Borosilicate (functional): Experimental dielectric data are reported by Alhaji (2024) [104]; mechanical and optical parameters by Abd-Elsatar (2024) [17]. CTE and hermeticity derive from standardized borosilicate data [61,108,115]. Fused Silica: CTE and optical transmittance are benchmark values [24,105,106]. Other parameters are system-level averages from Shelby (2020) [61] and Varshneya (2019) [60]. Aluminosilicate (technical): Measured data from Nunes (2025) [86]. Starred parameters are standardized ranges [60,61]. Hybrid and Low-Alkali systems: Values correspond to representative compositions from Jiang (2025), Lu (2019), and Wang (2025) [26,27,28] for phosphate- and aluminosilicate-based hybrids used in high-integrity or hermetic encapsulation. Data coverage: Even for the functional glass families listed, experimental data remain discontinuous and partly derived from industrial datasheets and standards; values represent consolidated reference ranges rather than complete datasets. Terminological scope: The borosilicate and aluminosilicate families reported here correspond to high-purity, low-alkali functional glasses for optical, electronic, and hermetic uses. They differ from the Type I/III borosilicate and high-alumina soda–lime (aluminosilicate) container glasses summarized in Table 2. Asterisk (*) notation: An asterisk indicates values taken from standardized ranges or manufacturers’ datasheets [60,61,105,108] rather than directly measured in the cited experimental studies. Permittivity and dielectric loss values are included only where consistent data are available. Values marked as (lit.) derive from the scientific literature analyzed in this study [46,49,82,104]. Values marked as (std.) derive from standard industrial datasheets [105,106] and reference texts [60,61]. Values marked as (est.) represent consolidated ranges for that glass family when direct measurements are not available in the analyzed literature.

## 3. Properties-Based Performance of Packaging Glass Families

The technical performance of packaging glass derives from the interplay between network structure, defect populations, and thermal–mechanical processing, which together determine stiffness, fracture resistance, thermal balance, chemical stability, and optical–barrier behaviour [13,16,52,53,54,56].

In this section, these property sets are analyzed in a comparative way to clarify how compositional features, network organization, modifier content, and processing conditions control the performance of the main packaging glass families.

**Figure 9:**
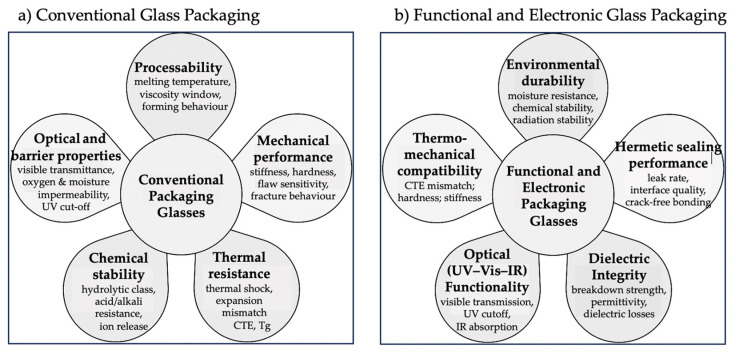
Property domains governing the technical performance of packaging glass families. (**a**) Conventional packaging glasses (soda–lime, borosilicate, aluminosilicate, cullet-rich recycled) are described by mechanical strength, thermal–chemical stability, chemical durability, optical/barrier performance, and processability, which collectively determine their suitability for food, beverage, cosmetic, and pharmaceutical containers. (**b**) Functional and electronic packaging glasses (low-alkali borosilicate, fused silica, aluminosilicate, boro-aluminosilicate, phosphate-modified hybrids and related functional compositions) are defined by hermeticity, dielectric integrity, optical functionality in the UV–visible–IR range, thermo-mechanical compatibility, and environmental durability, reflecting their role in microelectronics, photonics, diagnostics, and advanced encapsulation systems.

Figure 9 summarizes the performance domains adopted in this analysis, distinguishing the mechanical, thermal, chemical, optical–barrier, and processability requirements of conventional container glasses (Figure 9a) from the additional hermetic and dielectric constraints that characterize functional and electronic packaging architectures (Figure 9b).

**Figure 10:**
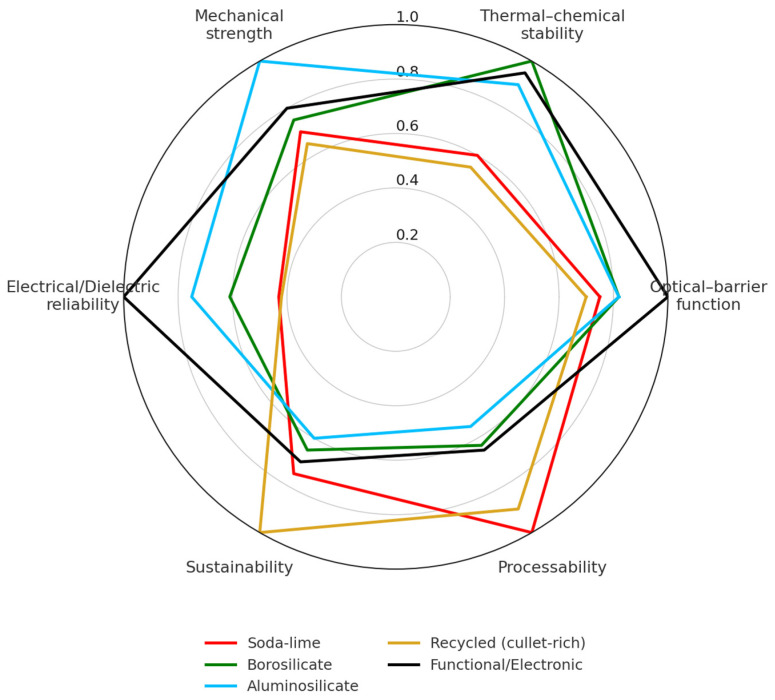
Normalized radar chart comparing the performance of soda–lime, borosilicate, aluminosilicate, cullet-rich recycled glass, and functional/electronic glass families across six domains: optical–barrier function, thermal–chemical stability, mechanical strength, electrical/dielectric reliability, sustainability, and processability. Scores are normalized to the best-performing composition in each domain (value = 1). Data derived from Table 2 and Table 3; normalization follows the axis-based method described in Section 3.

On this basis, and using the quantitative values reported in Table 2 and Table 3, Figure 10 provides a normalized comparison across the same domains for all glass families considered. Each axis in Figure 10 represents a composite indicator obtained by scaling the relevant properties to a 0–1 range and averaging them, then normalizing so that the best-performing family reaches a value of 1 on that axis.

In conventional food, beverage, cosmetic, and pharmaceutical packaging, these combinations of mechanical, thermal, chemical, and optical–barrier properties govern reliability during handling, filling, sterilization, and long-term exposure to products [55,116].

In functional and electronic packaging, the dominant requirements shift toward hermetic sealing, dielectric integrity, optical precision, and thermomechanical compatibility with silicon, metals, and microfabricated substrates [24,25,26].

**Figure 11:**
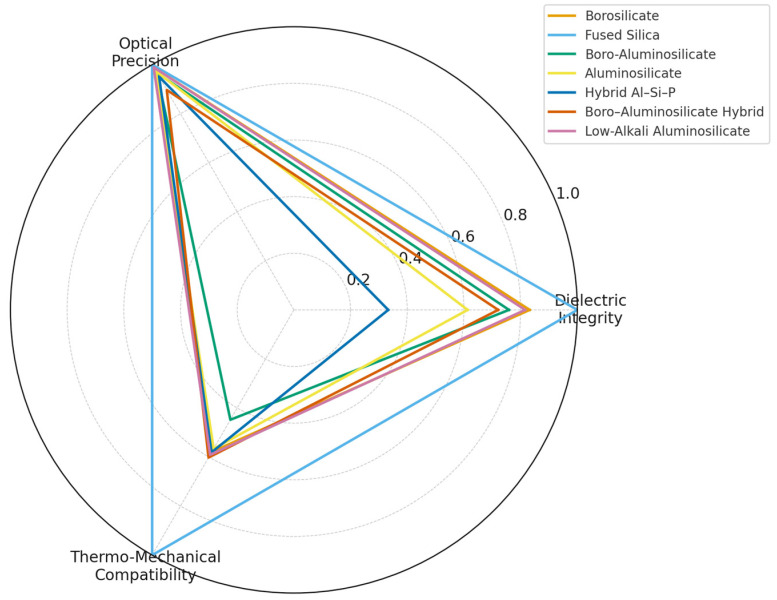
Normalized comparison of functional and electronic packaging glass families across three composite domains—dielectric integrity, optical precision, and thermo-mechanical compatibility. Each axis is scaled between 0 and 1, with 1 corresponding to the best-performing material in that domain. Dielectric integrity combines dielectric strength and loss tangent (tan δ), optical precision reflects visible transmittance (400–700 nm), and thermo-mechanical compatibility integrates CTE mismatch and surface hardness. Values are derived directly from the quantitative ranges reported in Table 3. Hermeticity and environmental durability are not included in this plot because available data are insufficiently homogeneous for normalization across all families; their comparative analysis is therefore addressed qualitatively in Section 3.2.1 and Section 3.2.5.

The quantitative values reported in Figure 10 and Figure 11 are intended as a comparative and illustrative representation of the relative performance of different packaging-glass families across multiple functional domains. These figures do not report absolute material properties but synthesize literature-supported indicators into a unified visual framework to highlight trends, trade-offs, and design constraints relevant to packaging applications.

The domains considered include mechanical integrity, thermo-mechanical compatibility, chemical durability, optical functionality, dielectric performance, hermetic sealing capability, and circularity/sustainability indicators. Each domain is defined on the basis of the material attributes discussed throughout Section 2.1, Section 2.2, Section 2.3, Section 2.4 and Section 2.5 and is supported by the quantitative ranges and qualitative evidence summarized in Table 1, Table 2 and Table 3 and in the corresponding subsections.

For each domain, representative indicators commonly reported in the literature were selected. For example, thermo-mechanical compatibility combines coefficients of thermal expansion, elastic modulus, and surface hardness; dielectric performance integrates dielectric strength, permittivity, and dielectric losses; optical functionality reflects visible transmittance; hermetic performance is evaluated on the basis of reported leak-rate ranges for representative sealing systems; and circularity/sustainability indicators consider recyclability, cullet compatibility, and process-related constraints. When multiple indicators contributed to a single domain, they were combined qualitatively or semi-quantitatively to reflect their relative influence.

To enable comparison across heterogeneous properties and material families, all indicators were normalized on a 0–1 scale. For each domain, a value of 1 corresponds to the best-performing glass family within the comparison set, while 0 corresponds to the lowest relative performance. Intermediate values represent proportional performance within the observed literature range. This normalization does not imply absolute ranking or intrinsic material limits but provides a consistent basis for cross-domain visualization.

The resulting indices, shown in Figure 10 and Figure 11, are therefore intended to support comparative interpretation and design-oriented discussion rather than precise quantitative ranking. The methodology and its limitations are explicitly acknowledged to ensure transparent and reproducible interpretation of the figures.

Building on the quantitative values reported in Table 2 and Table 3 and on the normalized comparison shown in Figure 10, the following subsections provide an interpretative discussion of how structural features, modifier content, and processing conditions map onto these performance domains. Section 3.1 and Section 3.2 examine these two domains separately, focusing on the structure–property relationships that underpin their mechanical, thermal, chemical, optical, and processing behaviour.

### 3.1. Conventional Packaging Glasses

Conventional packaging glasses—soda–lime, borosilicate, aluminosilicate, and cullet-rich recycled compositions—exhibit distinct performance profiles that reflect differences in network connectivity, modifier distribution, and defect control during forming and annealing. The comparative framework introduced in Figure 9 and Figure 10 provides the basis for interpreting these trends, linking the quantitative values of Table 2 and Table 3 to the structural and processing features that govern the behaviour of each family.

Soda–lime glass combines moderate network polymerisation with relatively high modifier content, which enhances melt fluidity and processability but limits thermal resistance and increases susceptibility to stress-driven chemical reactions. Borosilicate compositions reduce non-bridging oxygen fractions and expand the network through B–O–Si linkages, resulting in lower thermal expansion, improved chemical durability, and reduced thermal-shock sensitivity. Aluminosilicates display the highest degree of network connectivity among conventional families, with Al-rich tetrahedral units providing high stiffness and strength but also increasing viscosity and narrowing the forming window. Recycled cullet-rich glasses retain the fundamental behaviour of soda–lime systems, although variations in batch fining, residual defects, and redox state may influence melting behaviour, bubble populations, and surface quality.

In the following Section 3.1.1, Section 3.1.2, Section 3.1.3, Section 3.1.4 and Section 3.1.5, these structure–property relationships are examined across the five performance domains of mechanical behaviour, thermal response, chemical durability, optical–barrier performance, and processability, highlighting the mechanisms that determine why each glass family occupies its specific region in the normalized property space of Figure 10.

#### 3.1.1. Physical and Mechanical Properties

Mechanical behaviour in conventional packaging glasses arises from the combined effects of network polymerisation, modifier distribution, atomic packing density, and the flaw populations generated during forming and annealing. Density offers a first indication of network compactness: aluminosilicate glasses, dominated by Si–O–Al linkages, display the highest densities because [AlO_4_]^−^ tetrahedra increase network connectivity and reduce the fraction of non-bridging oxygens, resulting in a tightly packed structure [16,53]. Soda–lime glasses contain larger proportions of alkali and alkaline-earth modifiers, which open the network, increase free volume, and lower overall compactness, consistent with their higher reactivity and reduced mechanical rigidity [56]. Borosilicate glasses occupy an intermediate region: the coexistence of trigonal BO_3_ and tetrahedral BO_4_ units lowers packing density while conferring enhanced structural flexibility and reduced residual stress upon cooling [54]. This gradation in network compactness—aluminosilicate > borosilicate > soda–lime—is consistent with their mechanical hierarchy and provides the structural basis for the mechanical distribution observed in Figure 10.

Elastic stiffness and hardness scale with network connectivity. Aluminosilicate glasses reach the highest modulus because [AlO_4_]^−^[AlO_4_]^−^ tetrahedra introduce additional cross-links and lower the fraction of non-bridging oxygens, leading to a rigid, highly constrained network [16,53]. Borosilicate glasses show intermediate stiffness but benefit from reduced residual stress during cooling owing to their lower thermal expansion and more flexible mixed borate–silicate network [54]. Soda–lime glasses exhibit the lowest stiffness among conventional packaging compositions because their higher modifier content disrupts the network and increases non-bridging oxygens [56], although their mechanical performance remains adequate for high-throughput container production, where dimensional control, automated handling, and impact resistance are the primary constraints [55,116].

Fracture behaviour in conventional packaging glasses reflects both the intrinsic strength of the silicate network and the severity of surface flaws introduced during forming and handling. Aluminosilicate glasses, processed at higher viscosities, tend to be less susceptible to bubble entrapment and severe surface damage because their stiffer melts are less prone to shear imprinting and mould-induced defects [53]. Soda–lime glasses, formed at lower viscosity under high-throughput conditions, are more sensitive to micro-defects generated during mould contact, shear loading, and rapid cooling, which act as critical flaw sites and reduce effective strength [13,52]. Borosilicate glasses benefit from a favourable balance of viscosity, thermal expansion, and rigidity, which mitigates residual stresses and limits flaw severity, even though the intrinsic brittleness of silicate networks remains unchanged [16,54]. Cullet-rich compositions generally mirror the fracture behaviour of soda–lime glasses, but variations in fining efficiency, redox conditions, and recycled-derived inclusions can modify flaw populations and lead to broader scatter in fracture strength [13,56].

Overall, mechanical performance results from the interplay between intrinsic network rigidity, atomic packing density, and extrinsic flaw statistics, which together explain the distribution observed in Figure 10: aluminosilicates dominate the mechanical axis, borosilicates occupy an intermediate and relatively stable region, whereas soda–lime and cullet-rich compositions populate the lower range due to their more open structure and higher defect sensitivity.

#### 3.1.2. Thermal Properties and Shock Resistance

Thermal behaviour and shock resistance in conventional packaging glasses derive from network topology, modifier content and the degree of structural polymerisation, which together control thermal expansion and stress development under temperature gradients. Shock resistance denotes the ability of a glass to withstand rapid temperature changes without fracture, a performance that depends on both the magnitude of thermally induced stresses and the rate at which the network can relax them.

Borosilicate glasses display the lowest thermal expansion among conventional packaging families, a behaviour linked to the combined presence of trigonal BO_3_ and tetrahedral BO_4_ units and to the high network polymerisation reported for commercial borosilicates [54]. Their low fraction of non-bridging oxygens limits stress development during temperature excursions, providing the high thermal-shock tolerance widely reported for pharmaceutical and laboratory applications [54,80].

Soda–lime glasses show the highest expansion coefficients (Table 2), reflecting their higher modifier content and more open network architecture. The larger population of non-bridging oxygens increases local mobility and raises CTE values, reducing resistance to rapid temperature changes; reported failure modes under thermal stress in soda–lime containers are consistent with this behaviour [16]. Thermal-shock susceptibility is further amplified when residual stresses from fast forming or insufficient annealing are present [52].

Cullet-rich recycled glasses largely inherit the thermal expansion of soda–lime matrices (Table 2), but compositional variability and defect populations associated with recycled feedstocks can broaden the range of thermal-shock responses [13].

Aluminosilicate glasses display intermediate expansion values (≈3.8–4.2 × 10^−6^ K^−1^), positioned between borosilicate and soda–lime compositions (Table 2). The incorporation of [AlO_4_]^−^ tetrahedra increases network rigidity, while the alkali modifiers required for charge compensation reintroduce moderate structural mobility, preventing expansion from reaching the low levels typical of borosilicates [53]. As a result, aluminosilicates combine high stiffness with a moderate ability to accommodate temperature gradients.

Shock resistance reflects the combined effects of expansion, heat-transport properties, and stress-relaxation behaviour. These mechanisms explain the distribution shown in Figure 10: borosilicates dominate the thermal domain due to their very low expansion, aluminosilicates occupy an intermediate region, and soda–lime and cullet-rich compositions fall in the lower range because of their higher CTE and reduced tolerance to thermally driven stresses.

#### 3.1.3. Chemical Stability and Corrosion Resistance

Chemical stability and corrosion resistance in conventional packaging glasses arise from the interplay between network polymerisation, alkali mobility, and the distribution of non-bridging oxygens (NBOs), which together control susceptibility to hydrolysis, proton-exchange reactions, and surface dissolution. Chemical stability refers to the intrinsic resistance of the silicate network to hydrolysis and structural modification, whereas corrosion resistance describes the surface response to dissolution, ion exchange, and leaching under specific environmental conditions. Their combined effect defines each family’s position along the chemical axis of Figure 10 and determines long-term stability in contact with beverages, pharmaceuticals, detergents, and humid environments.

Soda–lime glasses exhibit the lowest intrinsic chemical stability among the conventional families because the high concentration of alkali modifiers and NBO-rich sites facilitates proton exchange, accelerates early-stage hydration, and promotes depolymerisation under both acidic and alkaline media [16,56]. These mechanisms support ion-exchange reactions such as Na^+^ ↔ H^+^, leading to the formation of hydrated silica layers and increased network disruption.

Corrosion resistance is similarly limited. High alkali mobility favours leaching in detergents, acidic beverages, and CO_2_-rich environments, while humid storage promotes the formation of alkali-carbonate blooms and heterogeneous surface films [16]. These effects explain the lower chemical position of soda–lime glass in Figure 10 and correspond to the hydrolytic Classes II–III reported in Table 2.

Borosilicate glasses achieve markedly higher chemical stability due to their low alkali content and their mixed B–O–Si network, where BO_3_/BO_4_ units suppress NBO formation and reduce the susceptibility of the network to proton attack [54]. The presence of strong B–O–Si linkages increases resistance to hydrolysis and limits the mobility of modifying cations.

Corrosion proceeds through a slow, surface-controlled mechanism: the hydrated layer grows gradually without significant bulk dissolution, providing excellent resistance in neutral and mildly aggressive solutions and maintaining stability during sterilization cycles and thermal shocks typical of pharmaceutical packaging [16]. This behaviour is consistent with hydrolytic Class I performance and explains the upper placement of borosilicates on the chemical axis of Figure 10.

Aluminosilicate glasses combine high chemical stability with controlled corrosion behaviour because [AlO_4_]^−^ tetrahedra introduce strong cross-linking and reduce the mobility of alkali modifiers [53]. The Si–O–Al network is intrinsically more resistant to hydrolysis than soda–lime systems, and the reduced NBO fraction suppresses proton-driven depolymerisation.

Corrosion processes are limited but may involve localized leaching of charge-compensating cations when they are only weakly integrated into the network. Nevertheless, resistance to both acidic and alkaline media remains substantially higher than in soda–lime glass and only slightly inferior to borosilicates, resulting in an intermediate—but robust—position along the chemical axis of Figure 10.

Cullet-rich recycled glasses retain the chemical characteristics of soda–lime matrices but exhibit greater variability due to fluctuations in cullet composition, redox state, sulphate residues, and recycled-derived inclusions [13]. These heterogeneities can influence intrinsic chemical stability, modifying early hydration kinetics and altering the local propensity for proton exchange.

Corrosion resistance is therefore broader in its response range. In some high-quality cullet streams, behaviour approaches that of standard soda–lime glass, whereas in less controlled feeds, surface dissolution and weathering can be accelerated, particularly during the first stages of exposure [56]. These differences explain the wider spread of cullet-rich systems along the lower region of the chemical axis in Figure 10.

Across these four families, the combined influence of alkali mobility, NBO concentration and network cross-linking defines a consistent hierarchy: borosilicate > aluminosilicate > soda–lime ≈ cullet-rich. This ordering is fully consistent with the chemical domain of Figure 10 and with the hydrolytic classifications and dissolution behaviours summarized in Table 2.

#### 3.1.4. Optical and Barrier Properties

Optical and barrier properties in conventional packaging glasses originate from the combined effects of electronic absorption, network purity, and the structural compactness that governs the propagation of photons and the reactivity of the near-surface layer. In the context of Figure 10, barrier refers to spectral shielding, namely, UV cut-off, chromophore-induced attenuation, and suppression of defect-related absorption—together with the chemical stability of the surface under light, humidity, or mildly corrosive environments. It does not refer to gas permeability, which is negligible across all glass families. This behaviour arises from the fully cross-linked, non-porous Si–O network: the absence of continuous free-volume pathways and the very high activation energies required for atomic or molecular diffusion (several hundred kJ·mol^−1^) prevent oxygen, nitrogen, water vapour, or organic molecules from migrating through the bulk under packaging-relevant conditions.

Soda–lime glasses exhibit high visible transparency, but their Fe^2+^/Fe^3+^ balance and the presence of minor chromophores (including Cr-bearing impurities) introduce characteristic absorption near the UV cut-off [16]. Their relatively open structure, rich in non-bridging oxygens, shows slightly higher free volume and greater susceptibility to early-stage surface hydration, which may transiently influence barrier performance at the glass–environment interface [56]. In coloured variants, these same chromophores act as optical barriers: Fe–S clusters in amber and Fe–Cr combinations in green compositions enhance UV attenuation, improving protection for light-sensitive formulations.

Borosilicate glasses display superior optical stability due to their high-purity raw materials, low alkali content, and minimal transition-metal impurities. The resulting network reduces light absorption arising from network defects or impurities and produces a stable, well-defined UV cut-off with lower variability than in soda–lime compositions [54]. Their compact structure and reduced surface reactivity ensure consistent optical performance during sterilization and storage, placing them at the upper end of the optical–barrier domain in Figure 10.

Aluminosilicate glasses combine moderate transparency with enhanced resistance to surface alteration. Their dense network, dominated by [AlO_4_]^−^ tetrahedra, limits alkali mobility and restricts hydration-layer formation, improving stability under light- and humidity-driven exposure [53]. Although their chromophore content is generally low, minor compositional variations can introduce limited spectral shifts. Overall, they occupy an intermediate position in Figure 10, with optical stability superior to soda–lime but below that of high-purity borosilicates.

Cullet-rich recycled glasses largely reproduce the optical behaviour of soda–lime matrices but may exhibit a wider performance range due to fluctuations in redox state, chromophore distribution, and residual inclusions derived from mixed-colour feedstocks [13]. These factors broaden near-UV absorption and may modulate early-stage surface hydration, although bulk transmission and gas impermeability remain fully consistent with conventional soda–lime glass.

Overall, the optical–barrier axis of Figure 10 reflects the interplay between intrinsic network purity, chromophore content, and surface stability. Borosilicates occupy the upper region due to their clean electronic spectra and chemically robust surface; aluminosilicates form a stable intermediate class; and soda–lime and cullet-rich glasses lie in the lower range because of chromophore-driven absorption and higher susceptibility to early surface alteration. These trends demonstrate that optical–barrier performance originates from the same network-controlled mechanisms—purity, modifier mobility, and near-surface reactivity—that define the broader functional hierarchy among packaging glasses.

#### 3.1.5. Processability and Lightweighting

Processability in conventional packaging glasses depends on the interplay between network structure, modifier content, melt viscosity, fining behaviour, and stress development during forming and cooling. These factors collectively determine the ease with which glass can be melted, homogenized, shaped, and stabilized in high-throughput manufacturing environments, and explain the distribution observed along the processability axis of Figure 10.

Soda–lime glasses exhibit the highest processability among the conventional families. Their open silicate network, enriched in alkali and alkaline-earth modifiers, lowers melt viscosity across the working range (10^3^–10^6^ Pa·s) enabling efficient fining and homogenisation [69]. High-cullet batches further reduce melting enthalpy and stabilize furnace redox conditions, improving melting and refining efficiency [13]. The broad viscosity window supports blow–blow and press–blow routes, enabling lightweighting up to 30–35% in narrow-neck press-and-blow operations [7,8]. However, low-viscosity forming increases susceptibility to shear-related defects, mould imprinting, and bubble entrainment [52], while rapid cooling combined with high thermal expansion promotes residual stresses if annealing is not properly controlled [13].

Borosilicate glasses display a narrower processability range due to higher melt viscosity and working temperatures. Tubing conversion rather than blow–blow forming is therefore the dominant industrial route for Type I pharmaceutical packaging [3,6]. Thermal-processability studies confirm that small compositional shifts—such as B_2_O_3_/alkali ratio or alkaline-earth substitution—significantly affect softening temperature and working interval [117], reducing forming flexibility but supporting stable flame-working for vials, cartridges and ampoules [54]. Borosilicates therefore occupy an intermediate position on the processability axis of Figure 10: less forgiving than soda–lime in high-speed container forming, but highly reliable in tubular geometries requiring precise thermal control.

Aluminosilicate glasses show the lowest processability among the conventional families. High Al_2_O_3_ levels increase network connectivity and substantially raise melt viscosity and softening temperature, narrowing the forming window [53]. In alkali-bearing compositions used for chemically strengthened vials and diagnostic cartridges, thermal pre-conditioning prior to ion-exchange influences medium-range structure and residual-stress development [87]. These constraints restrict aluminosilicate forming to tightly controlled, high-value pharmaceutical and diagnostic packaging, where stringent thermal management and precise dimensional control are required to ensure mechanical reliability [3]. Within Figure 10, these compositions occupy the lower end of the processability axis.

Cullet-rich recycled glasses largely retain the processability of soda–lime systems because cullet has the same oxide composition and enters the melt in an already amorphous state. High-cullet batches preserve viscosity ranges compatible with press-and-blow and narrow-neck press-and-blow forming, and reduce melting energy demand since pre-melted cullet requires less thermal input than crystalline raw materials [69]. However, processability becomes increasingly conditioned by cullet quality: colour cross-contamination, refractory particles, coatings, and fines influence fining efficiency, bubble populations, and forming stability [66]. Substitutability models indicate that only about 83% of cullet can effectively replace virgin materials without degrading melt behaviour, primarily due to impurity-driven variability [12]. Industry-4.0/5.0 approaches integrating advanced sorting, online defect detection, and AI-assisted furnace control help stabilize forming at high-cullet fractions [90]. As a result, cullet-rich soda–lime glasses cluster near conventional soda–lime on the processability axis, but their position is ultimately bounded by cullet quality and defect management rather than intrinsic rheology.

#### 3.1.6. Property–Driven Positioning of Glass Families in Packaging Applications

The combined mechanical, thermal, chemical, optical–barrier, and processing behaviour of conventional packaging glasses determines not only their position within the performance landscape of Figure 10 but also their suitability for specific packaging functions. The configuration of each silicate network—whether open, boron-modified, or alumina-stiffened—maps directly onto the constraints of food, beverage, cosmetic, and pharmaceutical packaging, where stress exposure, temperature swings, chemical aggressiveness, and visual requirements differ sharply [60,61].

Soda–lime glasses, with their broad forming window and high processability, remain the backbone of mass-market packaging [6]. Their moderate stiffness and higher thermal expansion align well with applications such as carbonated-beverage bottles, standard beer bottles, food jars, and wide-mouth containers, where mechanical loads are dominated by filling, capping, and handling rather than extreme thermal or chemical stress [13,56]. Their optical response, influenced by Fe-related chromophores, is suitable for coloured bottles—amber, green, or flint with coatings—where UV protection is achieved through pigmentation rather than intrinsic network design. Their cost–performance balance and excellent compatibility with high-throughput forming explain their dominance in global food and beverage markets.

Borosilicate glasses address a different performance space. Their low thermal expansion, high chemical durability, and optical stability make them indispensable wherever sterilization, thermal cycling, or chemical purity are essential to product safety [48,62]. This includes pharmaceutical vials, prefillable syringes, cartridges for injectables, and laboratory ampoules, as well as high-end perfume flacons that benefit from the material’s superior clarity and stable colour under UV exposure. Their higher viscosity complicates shaping, but these compositions occupy premium segments where reliability, not throughput, governs material selection.

Aluminosilicate glasses occupy a more specialized yet technically compelling region. Their highly polymerised networks support thin-walled geometries capable of sustaining substantial mechanical loads and resisting abrasion during transport and repeated handling [17,64]. These attributes enable applications such as impact-resistant jars, portable product housings, and premium cosmetic containers where scratch resistance and mechanical robustness are decisive. Although their high softening temperatures and narrow forming margins restrict widespread adoption, aluminosilicates provide mechanical advantages where weight reduction or enhanced durability re-define packaging performance. They also form a natural link toward the functional families discussed in Section 3.2, where high network connectivity and dielectric stability become dominant design attributes.

Cullet-rich glasses follow the behaviour of soda–lime matrices but introduce variability associated with recycled feedstock. Colour uniformity, defect populations, and surface stability limit their use in high-clarity cosmetic bottles or pharmaceutical primary packaging, which generally require virgin compositions [66]. Nonetheless, cullet-rich systems remain central to food and beverage packaging—wine bottles, beer bottles, food jars—where their mechanical and chemical performance is sufficient, and their environmental advantages are decisive [12].

Across these families, the interplay between structure, processability, and performance determines whether a glass composition can support the demands of sterilization, carbonation pressure, optical purity, flavour protection, or mechanical robustness. These relationships define the functional envelope of conventional packaging and establish the conceptual contrast with the materials examined in Section 3.2, where the dominant requirements shift from container reliability to hermetic sealing, dielectric integrity, and micron-level optical and structural precision.

### 3.2. Functional and Electronic Packaging Glasses

Functional and electronic packaging glasses comprise high-purity borosilicates, fused silica, technical aluminosilicates, aluminosilicate–phosphate hybrids, and mixed borosilicate–aluminosilicate systems. Unlike conventional container glasses, their design priorities are aligned with the functional domains illustrated in Figure 9b—hermetic sealing behaviour, dielectric integrity, thermo-mechanical compatibility with silicon and metals, optical precision, and environmental durability—rather than structural robustness or large-scale formability. These requirements reflect their use in device-level architectures, including wafer-level MEMS encapsulation, IR and UV optical windows, alkali-free dielectric layers, microfluidic substrates, and through-glass-via (TGV) interposers for microelectronics.

The quantitative ranges reported in Table 3 highlight the diversity of these systems and capture property combinations not achievable in conventional soda–lime, borosilicate, or aluminosilicate glasses. To provide an interpretable overview of their relative behaviour, Figure 11 compares the main functional glass families across three composite domains—dielectric integrity, optical precision, and thermo-mechanical compatibility—each normalized to the best-performing material on its axis. These indices were derived directly from the values in Table 3: dielectric integrity combines breakdown strength and dielectric losses, optical precision reflects visible transmittance, and thermo-mechanical compatibility incorporates CTE mismatch and surface hardness.

This normalized comparison provides the framework for the following Section 3.2.1, Section 3.2.2, Section 3.2.3, Section 3.2.4 and Section 3.2.5, which examine each functional domain in detail, clarifying how compositional design, network topology, and interfacial behaviour govern the performance of functional and electronic packaging glasses.

#### 3.2.1. Hermeticity

Hermetic sealing performance in functional packaging glasses reflects the ability to form crack-free, chemically compatible, and gas-tight interfaces with metals, silicon, or other glasses. It is not a bulk material property, but the outcome of how glass composition, viscosity at the sealing temperature, redox stability, wetting behaviour, and thermomechanical compatibility interact during the joining process. The leak-rate values in Table 3 (typically in the 10^−8^–10^−9^ mbar·L·s^−1^ range) therefore represent system-level sealing performance rather than an intrinsic characteristic of the glass.

Low-temperature frit glasses—based on Bi_2_O_3_–B_2_O_3_–ZnO or related modified borate systems—exhibit controlled softening between 350 and 500 °C, allowing sealing at temperatures compatible with MEMS, infrared sensors, micro-optical assemblies, and micro-batteries [49]. Their redox stability suppresses bubble formation and reduces the entrapment of volatile species, leading to leak rates in the 10^−8^–10^−9^ mbar·L·s^−1^ range during vacuum packaging [43]. Borosilicate and fused-silica wafers, used in anodic bonding and high-purity optical encapsulation, routinely achieve similar hermeticity (≤10^−9^ mbar·L·s^−1^), mainly due to their low alkali content, stable surface chemistry, and strong electrostatic bonding to silicon (Tanaka 2014) [40]. Aluminosilicate and boro-aluminosilicate glasses also reach leak rates of 10^−8^–10^−9^ mbar·L·s^−1^ in laser-assisted or glass-frit hybrid sealing, provided that thermal expansion mismatch is kept below the cracking threshold [82].

In Figure 9b, hermeticity is therefore represented as a performance domain that reflects the capacity of a glass family to support vacuum-tight encapsulation, rather than a single intrinsic material parameter. By contrast, neither Figure 10 nor Figure 11 includes hermeticity among their axes, because these comparative plots are restricted to material-level indicators (mechanical, thermal–chemical, optical, processability, sustainability, dielectric reliability). The leak-rate values reported in Table 3 thus provide a practical benchmark of sealing performance across functional glass families, while the discussion in this subsection clarifies the compositional and interfacial factors that enable such levels of hermeticity.

#### 3.2.2. Dielectric Performance

Dielectric integrity describes the ability of a glass to sustain electric fields without breakdown, maintain stable permittivity, and minimize dielectric losses over the relevant operating frequency range. Dielectric performance is primarily a material property of the glass network, governed by bond polarizability, defect populations, alkali mobility, and structural relaxation processes. The electrical parameters summarized in Table 3—permittivity ε′, dielectric loss tan δ, and dielectric strength—define the dielectric domain represented in Figure 11 [24,60,61,86,104].

At the structural level, permittivity increases with the polarizability of the network-forming units and with the ease of distorting local bonding configurations under an electric field. Silica-rich glasses, dominated by strongly covalent Si–O bonds, display the lowest polarizability, whereas the incorporation of B_2_O_3_, Al_2_O_3_, P_2_O_5_, or heavy-metal oxides introduces more deformable bonds and mixed coordination environments that raise ε′ [41,49,112,113]. Network topology—specifically the balance between bridging and non-bridging oxygens, the connectivity of tetrahedral units, and the presence of charge-compensated [AlO_4_]^−^ groups—further modulates the electronic response [60,61].

Dielectric losses arise primarily from modifying-cation mobility and defect-related relaxation processes. Glasses with very low alkali content and high chemical purity contain few pathways for ionic motion and exhibit minimal structural or redox-related defects, resulting in extremely low loss tangents [105,106,108]. As alkali concentration increases, or as the network incorporates multivalent cations and more heterogeneous local environments, additional relaxation modes become active, broadening the frequency range over which energy is dissipated [82,114].

Breakdown strength reflects the resistance of a glass to the nucleation and growth of electrically driven defects. Dense, highly covalent networks with low defect concentrations sustain the highest breakdown fields, whereas the introduction of more polarizable units, heavier cations, or heterogeneous structural motifs increases local field amplification and lowers the breakdown threshold [26,86,102,104].

Figure 11 integrates these descriptors into a single normalized indicator, from which a consistent hierarchy emerges. Silica-rich borosilicate and fused-silica glasses occupy the upper region of the dielectric domain, combining strong covalency with minimal alkali mobility and low defect populations [24,108]. Boro-aluminosilicate and LAS-type hybrid glasses form an intermediate tier, where increased polarizability is partially offset by constrained modifier dynamics [41,113]. Technical aluminosilicate glasses, with higher fractions of charge-compensated [AlO_4_]^−^ units and occasional rare-earth species, shift toward the mid–upper part of the hierarchy [86,114]. Hybrid aluminosilicate–phosphate and Bi-containing low-temperature sealing glasses define the lower tier, where highly polarizable P–O and Bi–O units and greater structural heterogeneity broaden relaxation spectra and reduce breakdown strength [41,49].

#### 3.2.3. Optical Functionality in the UV–Visible–IR Range

Optical functionality in functional packaging glasses spans the ultraviolet (UV), visible, and infrared (IR) ranges and depends on their ability to maintain high transmittance, low absorption, and spectral stability across the operational wavelength window of micro-optical, photonic, and micro-electro-mechanical systems (MEMS) devices. These behaviours are controlled by network purity, the presence of non-bridging oxygens, electronic transitions associated with modifier oxides, and absorption features arising from heavy-metal constituents. Differences in UV, visible, and IR responses across the functional glass families define the “Optical Functionality” domain shown in Figure 9b. In Figure 11, only the visible region is compared, as it is the spectral interval for which quantitative and cross-family data are consistently available in Table 3.

Fused silica exhibits the highest optical purity. Its >99.8 wt% SiO_2_ composition and minimal defect population ensure transmittance exceeding 92% in the 400–700 nm range, with low absorption extending into the UV down to ~180–200 nm and into the near-IR up to ~2.5 µm [105,106]. The absence of multivalent oxides suppresses colour centres and electronic absorption bands, making fused silica the benchmark for both UV transparency and IR stability.

High-purity borosilicate glasses maintain similarly high visible transmittance (>90%) [17,104]. Their UV response is slightly less extended than fused silica due to boron-containing structural units, which introduce absorption below ~300 nm. In the near-IR, borosilicates retain low absorption up to ~2–2.5 µm, with losses increasing beyond this range because of modifier-related overtone bands.

Boro-aluminosilicate and technical aluminosilicate glasses show intermediate visible transmittance (≈88–91%) [102,114]. Their UV cutoff shifts toward longer wavelengths because of higher concentrations of network modifiers and Al–O–Si linkages, which introduce additional electronic transitions. In the IR, these compositions display stronger absorption in the mid-IR range due to overtones from modifier cations and more complex vibrational environments.

Hybrid aluminosilicate–phosphate and Bi–B–Si low-temperature sealing glasses have lower visible transmittance (≈80–90%) owing to heavy-metal oxides (Bi_2_O_3_, ZnO) and phosphate structural units, which introduce localized electronic transitions and elevate absorption in both the visible and near-IR [43,118]. Their UV transmittance is substantially reduced, and their IR attenuation becomes significant beyond ~1.2 µm, reflecting the strong electronic and vibrational contributions of heavy-metal constituents.

Taken together, these UV–visible–IR behaviours produce a coherent spectral ordering across functional glass families. Figure 11 condenses this spectrum of responses by comparing normalized visible transmittance: fused silica and high-purity borosilicate occupy the upper tier, boro-aluminosilicate and aluminosilicate compositions lie in an intermediate region, and heavy-metal-rich sealing glasses define the lower boundary. This visible-range comparison provides the quantitative backbone of the broader UV–visible–IR functionality discussed above, while UV cutoff and mid-IR absorption differences are evaluated qualitatively in the preceding paragraphs.

#### 3.2.4. Thermal–Mechanical Compatibility

Thermal–mechanical compatibility describes the ability of a functional glass to remain dimensionally stable and crack-free when bonded to silicon, metals, or ceramics and subjected to thermal excursions. This behaviour is controlled by the mismatch in CTE, the elastic modulus of the glass network, and the surface hardness, which governs resistance to imprinting and defect generation. While the thermal-mismatch stress scales approximately as σ∝E Δα ΔT, where σ is the thermally induced stress, E is the Young’s modulus of the glass, Δα is the CTE mismatch between the bonded materials, and ΔT is the temperature change, the onset of cracking or interfacial degradation also depends on the glass’s ability to suppress surface damage and micro-indentation during bonding and subsequent thermal cycling. The quantitative ranges of CTE, modulus, and hardness compiled for the main functional glass families in Table 3 define the thermo-mechanical domain recalled in Figure 9b, and their combined effect is summarized in the normalized CTE–hardness positioning shown in Figure 11. The structural origin of these differences can be traced to network rigidity, bond strength, and the role of modifiers. More highly polymerised Si–O–Si networks yield lower CTE and moderate stiffness, whereas the incorporation of more deformable units (e.g., B–O, P–O) or network modifiers increases thermal expansivity. The elastic modulus follows the strength and connectivity of the network-forming units, rising with rigid Si–O and Al–O tetrahedra and decreasing when heavier or less covalent species are present. Hardness mirrors this behaviour: densely cross-linked, strongly covalent networks resist local yielding, while glasses containing heavy-metal oxides or more heterogeneous bonding environments exhibit lower resistance to surface deformation. These structural factors provide a unified rationale for the inter-family differences visible in Table 2 and Table 3 and underpin the discussion below.

Fused silica occupies the most favourable region of the thermo-mechanical landscape. Its ultra-low CTE (0.5–0.55 × 10^−6^ K^−1^) and moderate elastic modulus (≈72 GPa) minimize thermal-mismatch stresses with silicon, metals such as Al or Cu, and ceramic substrates, while its high hardness limits surface deformation during bonding. These features explain its upper-tier position in Figure 11 and its broad compatibility across wafer-level architectures [40,43].

High-purity borosilicate glasses exhibit balanced thermo-mechanical behaviour, combining a low CTE (≈3.2–3.4 × 10^−6^ K^−6^) with a moderate modulus (≈47–48 GPa). The limited alkali content restricts stress-corrosion effects during thermal cycling, and the CTE remains well aligned with standard silicon packages. Their CTE–hardness combination situates borosilicates in the intermediate, stable region of Figure 11 [104].

Boro-aluminosilicate glasses exhibit slightly higher stiffness (≈68–75 GPa) and CTE values around 4.2 × 10^−6^ K^−1^. Their mixed-network structure provides good hardness and limits imprinting during bonding, but their higher CTE moves them modestly away from the silica match. As shown in Figure 11, they lie just above borosilicates in the normalized thermo-mechanical hierarchy [102].

Technical aluminosilicate glasses—including ion-exchange-strengthened variants—combine high hardness (≈6–7.7 GPa) with relatively high modulus (≈85–87 GPa) and moderate CTE values (≈3.5–4.5 × 10^−6^ K^−1^). The resulting thermo-mechanical stresses are higher than in borosilicates but remain manageable when compressive surface layers are present. Their elevated hardness moves them toward the upper-right region of Figure 11, reflecting their robust but less compliant behaviour under thermal cycling [25,114].

Hybrid aluminosilicate–phosphate and borosilicate–aluminosilicate compositions present intermediate modulus values (≈60–70 GPa) and tailored CTE ranges around 3.0–3.5 × 10^−6^ K^−1^. Their ability to flow and bond at 350–500 °C reduces residual stress during sealing, but their comparatively lower hardness limits their thermo-mechanical margin during subsequent handling or high-temperature cycling [26,28]. Their position in Figure 11 reflects this balance: moderate expansion mismatch with moderate surface resistance.

Low-alkali aluminosilicates, engineered for dielectric substrates and through-glass-via (TGV) architectures, combine high elastic modulus (≈80–90 GPa) with CTE values of 3.5–4.0 × 10^−6^ K^−1^ [27]. Their high stiffness amplifies mismatch-driven stresses and necessitates tight process control, although their high hardness supports dimensional stability during metallisation and cycling. This places them toward the rightmost region of Figure 11.

Taken together, the thermo-mechanical profiles of these glass families illustrate how CTE, stiffness, and hardness jointly determine compatibility with semiconductor, metallic, and ceramic architectures. Fused silica and high-purity borosilicates define the broadest compatibility window; boro-aluminosilicates and hybrids provide tailored but more constrained behaviour; and low-alkali aluminosilicates extend the domain toward high-stiffness substrates where mechanical compliance is limited.

#### 3.2.5. Environmental Durability

Environmental durability in functional and electronic packaging glasses reflects the ability of a composition to preserve structural, optical, and interfacial integrity under moisture exposure, chemical attack, and radiation fields. These behaviours originate from network polymerisation, modifier mobility, and the presence of heteroatomic units such as borates, aluminates, phosphates, or heavy cations, which jointly control hydrolytic resistance, surface hydration kinetics, and radiation-induced defect formation.

Fused silica exhibits the highest environmental durability among the functional families. Its fully polymerised SiO_2_ network (NBO ≈ 0) and absence of alkali modifiers strongly suppress hydrolysis, limit hydration-layer formation, and prevent ion migration, yielding minimal reactivity in wet or mildly aggressive environments [51]. Its radiation stability is also exceptional: the low concentration of intrinsic defects and the absence of multivalent cations minimize colour-centre formation, making fused silica suitable for high-power photonics, micro-optics, and radiation-rich electronic platforms.

High-purity borosilicate glasses also display strong environmental durability. Their reduced NBO fraction and low alkali content improve resistance to moisture-driven hydration and limit ion exchange, while the presence of B–O–Si linkages promotes surface stability under thermal and chemical cycling [104]. However, borosilicates are more susceptible than fused silica to radiation-induced boron-related defects, which can locally alter optical absorption or dielectric behaviour under high-dose exposure.

Boro-aluminosilicate glasses, containing mixtures of BO_3_/BO_4_ and AlO_4_ units, achieve balanced environmental performance. Their increased cross-link density moderates hydration rates and reduces susceptibility to alkaline attack, while ZnO/BaO additions can introduce heavier cations that influence radiation response [102]. These glasses generally retain surface integrity under humidity, but their environmental durability depends strongly on modifier distribution and redox state.

Aluminosilicate technical glasses offer high durability under moisture and moderate chemical exposure. The incorporation of AlO_4_^−^ tetrahedra increases network rigidity and reduces alkali mobility, limiting both hydration and ion-exchange-driven degradation [46]. Their environmental performance remains robust during thermal cycling and exposure to cosmetic or mildly acidic formulations, making them well suited for protective windows, sensors, and cover-glass applications. Radiation tolerance is moderate and composition-dependent, especially for high-Al_2_O_3_ formulations.

Hybrid aluminosilicate–phosphate glasses, although stable during low-temperature sealing, exhibit greater vulnerability to long-term moisture exposure. P–O–P and mixed P–O–M (M = Ca, Mg, Zn) linkages are less resistant to hydrolysis than Si–O–Si or Si–O–Al bonds, leading to higher ion release and surface degradation in humid or aqueous conditions [26]. These materials provide favourable processing windows but require encapsulation or barrier layers for prolonged environmental exposure.

Borosilicate–aluminosilicate hybrids display intermediate environmental durability. Their mixed-network structure reduces the dissolution tendency typical of phosphate-rich systems while offering better moisture resistance than alkaline boro-silicates, although their stability remains composition-dependent [28].

Low-alkali aluminosilicates, developed for dielectric substrates and TGV integration, demonstrate excellent resistance to moisture and chemical ageing. Their high cross-link density and minimal alkali content limit hydration, ion migration, and alkali-related surface reactions, ensuring dimensional stability during long-term operation in humid or chemically active environments [27]. Radiation stability is also comparatively good, with limited formation of defect centres.

Overall, the environmental-durability petalo of Figure 9b reflects the progressive transition from highly stable networks (fused silica, high-purity borosilicates) to compositions where non-silicate units or mobile modifiers introduce greater vulnerability to moisture and radiation. These mechanisms determine the relative positioning of the functional glass families in long-term applications where humidity, chemistry, and radiation exposure govern reliability, and explain why fused silica and low-alkali aluminosilicates dominate this domain, while hybrid phosphates require protective architectures or controlled environments.

#### 3.2.6. From Functional Domains to Electronic and Functional Packaging Architectures

The functional glass families discussed in Section 3.2.1, Section 3.2.2, Section 3.2.3, Section 3.2.4 and Section 3.2.5 are selected for packaging roles that go beyond simple structural containment. Their use in electronic and functional packaging is determined by the need to combine hermetic sealing, dielectric insulation, optical access, and thermo-mechanical compatibility with silicon, metals, and ceramics. These requirements define the functional domains of Figure 9b and explain why the composition–property relationships of the different glass systems must be resolved before assigning them to specific packaging architectures.

High-purity borosilicate glasses provide the baseline for hermetic caps and glass-to-metal seals in electronic and biomedical packaging. At wafer level, borosilicate capping wafers are bonded to silicon by anodic bonding or glass-frit bonding to realize hermetic MEMS packages and vacuum cavities for accelerometers, pressure sensors, micro-pumps, tactile and flow sensors [40,41,43,44]. In implantable neuromuscular microstimulators and other long-term medical devices, borosilicate capsules and glass-to-metal seals form the primary barrier between the electronics and body fluids, combining hermeticity, biocompatibility, and electrical insulation [119]. In all these cases, alkali-driven ion conductivity for anodic bonding, controlled CTE matching to silicon or metals, and stable dielectric behaviour under thermal and humidity cycling are the governing selection criteria.

Fused silica is employed where dielectric loss and dimensional stability are limiting factors in high-frequency packaging. It is used as a reference and alternative substrate for RF and microwave modules, as well as for TGV-based interposers in 3D wafer-level packaging of RF filters and sensor systems, where low permittivity, very low loss tangent, and high thermo-mechanical stability support miniaturized, low-loss interconnect architectures [44,48,50,51]. Its role is therefore primarily that of a low-loss, high-stability substrate or interposer in advanced electronic packaging rather than of a bulk containment material.

Boro-aluminosilicate glasses extend these functions toward engineered substrates for 3D integrated packaging and RF front-end modules. Aluminoborosilicate glass substrates with tailored alkaline-earth content are developed specifically for 3D integrated packaging, combining low dielectric constant, low loss, controlled CTE, and good processability for TGV formation and fine-line metallisation [45,46,120]. Hybrid borosilicate–aluminosilicate compositions follow a similar logic in 5G/6G packaging platforms, where they are optimized as low-loss electronic packaging substrates with appropriate CTE matching and high insulation resistance [48]. In these substrates, the dielectric domain of Figure 9b is coupled directly to routing density, insertion loss, and reliability in RF and 3D system-in-package architectures.

Aluminosilicate (ion-exchanged/technical) glasses contribute where mechanical robustness and surface integrity dominate. Ion-exchanged alkali aluminosilicate glass–ceramics exhibit high strength and damage tolerance and are exploited as mechanically demanding cover and substrate materials in electronic and sensor assemblies, where resistance to impact, abrasion, and thermal shock is required [82,86]. Lead aluminosilicate glasses are formulated as passivation layers for advanced chip packaging, electrically insulating and protecting Si-based power devices while remaining compatible with metallisation stacks and thermal cycling [118]. In these architectures, the mechanical and interfacial domains—strength, hardness, CTE matching, and surface quality—govern the deployment of aluminosilicate networks in protective and passivating roles.

Hybrid aluminosilicate–phosphate and related low-temperature phosphate or Bi-containing borate glasses enter packaging architectures primarily as sealing and structural materials processed at reduced temperature. Bi–B–Zn sealing glasses are designed to encapsulate fibre Bragg grating (FBG) sensors, providing a hermetic, mechanically compatible barrier around the optical fibre in high-temperature or chemically aggressive environments [101]. Bi_2_O_3_-rich borate and borophosphate glasses, together with BaO–B_2_O_3_–ZnO systems, are used as low-melting dielectric and sealing layers in power-electronics and display components, where they act as insulating, hermetic interlayers bonded to ceramic or metal substrates [98,99,118]. Glass-frit pastes derived from these families are the standard sealing materials for wafer-level hermetic packaging of MEMS and smart sensors, where the frit frame bonds glass or silicon caps to device wafers and forms vacuum or controlled-pressure cavities [41,43]. Low-temperature phosphate glasses have also been proposed as printable, chemically stable materials for multimaterial 3D printing of functional microfluidic devices, in which glass channels, sealing layers, and structural elements are co-fabricated with polymers or metals [24]. Here, low softening temperature, wetting behaviour, chemical durability, and long-term hermeticity are the decisive functional constraints.

Low-alkali aluminosilicate glasses close the sequence as substrates and TGV interposers in high-density electronic packaging. They are engineered to withstand TGV drilling and metallisation while maintaining low dielectric loss, high modulus, and controlled CTE, enabling dense vertical interconnects and stable RF performance in 2.5D/3D assemblies and sensor packages [45,46,50,51,120]. In these systems, the thermo-mechanical and dielectric domains must be balanced to guarantee both warpage control and high-frequency signal integrity under assembly and service conditions.

Across these glass families, the functional domains introduced in Figure 9b—hermeticity, dielectric integrity, optical access and thermo-mechanical compatibility—appear as direct selection criteria linking the compositions of Table 3 to specific packaging roles: high-purity borosilicates for hermetic MEMS caps and implantable housings, fused silica and low-alkali aluminosilicates for low-loss interposers and TGV substrates, aluminosilicates for mechanically robust covers and passivation layers, and hybrid phosphate- and Bi-containing glasses for low-temperature hermetic seals and microfluidic integration. This mapping between functional domains and application spaces clarifies the rationale for the property-based analysis in Section 3.2 and sets the basis for discussing design choices and sustainability constraints in the following section.

## 4. Circularity and Sustainability of Packaging Glass

The present section analyses packaging glass in terms of material circularity and environmental sustainability. The discussion focuses on container glass, for which recycling flows, cullet use, reuse schemes, and life-cycle indicators are documented in detail, while other packaging-relevant glass families are mentioned only where consistent information is available. Circularity and sustainability are examined separately so that the different aspects of container-glass performance can be evaluated more clearly.

Circularity describes how glass remains in a closed loop through collection, sorting, cullet quality, and bottle-to-bottle recycling, whereas sustainability concerns the environmental consequences of these flows, including energy demand, CO_2_ emissions, and the use of raw materials. In container glass the two dimensions are closely connected, because higher cullet contents directly lower melting energy and emissions, but they still refer to different aspects of performance. The subsections that follow examine these two dimensions for container glass and indicate where comparable evidence is missing for other packaging-relevant glass families.

**Figure 12:**
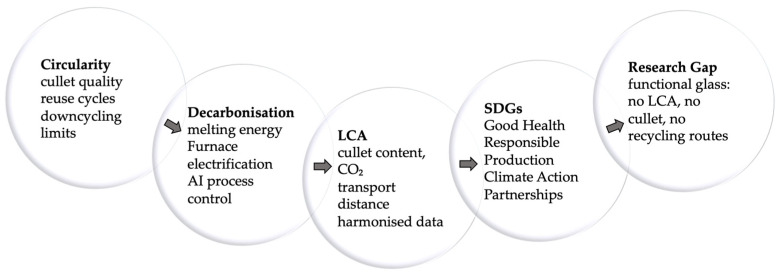
Roadmap of Section 4—Circularity and Sustainability of Packaging Glass. The figure summarizes the structure and logical progression of Section 4, from the analysis of circularity mechanisms in container glass to energy and decarbonisation strategies, life-cycle indicators, SDG alignment, and the documented research gap for functional and electronic packaging glasses. Each block highlights the key aspects discussed in its respective subsection.

A schematic overview of the structure and progression of this section is shown in Figure 12.

### 4.1. Circularity of Container Glass

Container glass represents one of the most effective circular material systems in the packaging sector, sustained by high collection rates, stable closed-loop recycling, and the ability to preserve chemical durability and optical clarity through multiple melting cycles. High-quality cullet is central to this performance: colour-sorted streams frequently exceed 90% purity in European systems [65], supporting efficient bottle-to-bottle recycling and reducing dependence on virgin raw materials. Multi-cycle remelting studies confirm that the structural and chemical properties of the glass network remain stable across repeated recycling loops, with no progressive degradation of durability or hydrolytic resistance [56].

Circularity is reinforced by well-established reuse models. Regional schemes demonstrate that even one reuse cycle delivers a favourable environmental balance relative to single-use bottles [93], while return systems supported by short-distance logistics enable 20–40 cycles before end-of-life collection [14]. These cycles coexist with recycling, forming a hybrid material loop where bottles transition from reuse to remelting depending on surface wear, contamination, and logistical conditions.

Closed-loop efficiency nevertheless depends strongly on material quality. Studies on cullet-rich batches highlight the sensitivity of high-percentage recycling to impurities, fines, organics, and colour mixing, all of which reduce the achievable cullet fraction or lead to partial downcycling [12,66]. Up-casting strategies—such as directing mixed cullet into higher-value applications—remain rare and technically constrained, confirming that the highest value retention is still achieved in conventional bottle-to-bottle loops [18,93]. The persistence of local inefficiencies, including uneven collection performance and losses in sorting facilities, limits the full recovery potential, but the overall system maintains one of the highest circularity levels among packaging materials.

### 4.2. Energy Demand and Decarbonisation of Container Glass

The melting process accounts for the majority of the environmental impact of container glass because it requires high temperatures and large amounts of energy. Across recent analyses, the melting stage consistently accounts for more than 70% of total CO_2_ emissions and primary energy use, largely due to the combustion of natural gas and the decomposition of carbonates in the batch [10,107]. This concentration of impacts places furnace operation at the core of all decarbonisation strategies.

Two complementary pathways emerge from the literature. The first concerns optimization of current melting technologies. Measures such as batch preheating, cullet enrichment, oxy-fuel firing, flue-gas heat recovery, and improved insulation deliver incremental but cumulative reductions in fuel demand. Digital furnace control, real-time thermal monitoring, and AI-assisted regulation have been shown to stabilize temperature profiles and reduce variability in energy consumption during melting campaigns [121]. Because cullet lowers the melting temperature and accelerates heat transfer, high-cullet batches enable additional efficiency gains beyond the direct reduction in process emissions [109,111].

The second pathway concerns progressive substitution of fossil energy sources. Hybrid-electric furnaces already demonstrate substantial reductions in direct emissions when powered by low-carbon electricity, while full electrification offers the largest theoretical climate benefit, provided that renewable electricity is available at adequate scale. Recent studies also consider hydrogen-assisted combustion as a transitional option for high-temperature zones of conventional furnaces, although its effectiveness depends on burner design, flame temperature, and the stability of the glass melt [107]. The use of low-carbon raw materials—such as decarbonated carbonates or recycled fines—further reduces process emissions by lowering the contribution of batch decomposition.

Decarbonisation trajectories for 2030–2050 therefore combine furnace electrification, increased cullet ratios, digital process optimization, and gradual substitution of fossil carbon in the batch. These developments reflect a convergence between industrial efficiency and environmental performance: improvements that reduce the energy required per tonne of molten glass simultaneously increase the sustainability of high-circularity recycling loops.

### 4.3. Life-Cycle Assessment Indicators for Container Glass

Life-cycle assessments consistently show that the environmental performance of container glass is strongly governed by recycling rates and cullet content. Typical global warming potential for standard bottle production ranges between 1.0 and 1.2 t CO_2_-eq per tonne of glass, with nearly linear reductions as cullet content increases [13,15]. For every 10% increase in cullet, studies report average decreases of ≈3% in energy demand and ≈ 2–3% in CO_2_ emissions, reflecting both the lower melting temperature of recycled glass and the avoided decomposition of carbonates.

Beyond climate-related indicators, LCA studies highlight the influence of logistics. Transport distance is a primary contributor to impact variability, especially for reuse and closed-loop recycling systems, where regional optimization determines whether the environmental balance remains favourable [65]. Collection efficiency and sorting quality further affect LCA outcomes by determining the achievable cullet fraction and the proportion of flows diverted to downcycled applications.

When compared with multilayer plastics or aluminum, glass performs favourably under scenarios where high-quality closed-loop recycling is maintained [15]. Recent work emphasizes the importance of harmonized datasets, digital twin models, and certified low-carbon batches to ensure consistency across assessments and support transparent reporting within sustainability frameworks [21].

### 4.4. SDG Alignment of Container Glass

The contribution of container glass to the UN Sustainable Development Goals reflects the combined effect of its inert chemistry, high circularity, and the ongoing decarbonisation of melting technologies. SDG 3 (Good Health and Well-Being) is supported by the chemical stability of glass, which prevents leaching and ensures safe contact with food, beverages, and pharmaceuticals [9]. SDG 12 (Responsible Consumption and Production) is reinforced by mature bottle-to-bottle recycling schemes, closed-loop cullet circulation, and the integration of reuse models within regional logistics systems [21].

Progress in furnace electrification, cullet enrichment, and hybrid-energy melting aligns with SDG 13 (Climate Action) by lowering CO_2_ emissions from both fuel combustion and raw-material decomposition [107]. Finally, efficient collection networks and coordinated industry–municipality partnerships contribute to SDG 17 (Partnerships for the Goals), reflecting the collaborative structure required to sustain high-quality recycling loops across regions [21].

These interactions position container glass as a material system where circularity and sustainability are mutually reinforcing, with policy-aligned improvements arising directly from increased cullet quality, decarbonised melting, and regionalised logistics. The documented SDG contributions are therefore tightly coupled to the performance of established recycling and reuse infrastructures.

### 4.5. Research Gap for Functional and Electronic Packaging Glasses

The available literature provides no established metrics of circularity or environmental sustainability for functional and electronic packaging glasses, including low-temperature sealing glasses, frit-based bonding systems, microelectronic encapsulation glasses, interposer/TGV substrates, and phosphate-based printable compositions. Existing studies focus on process performance, interfacial behaviour, and device reliability, but they do not report recycling routes, cullet streams, LCA datasets, or energy analyses specific to these materials [41,113,114]. Broader assessments of microelectronic packaging highlight the lack of upstream life-cycle inventories for advanced materials, confirming that environmental modelling remains limited by incomplete datasets.

As a result, the sustainability of functional packaging glasses can only be inferred indirectly from general trends in specialty-glass manufacturing—high melting energy, restricted recycling infrastructure, and limited material recovery. These constraints define a clear research gap and mark a divergence from container glass, where mature circularity mechanisms and robust LCA indicators allow performance to be quantified in a transparent and policy-aligned manner.

## 5. Regulatory Framework and Future Outlook

The regulatory framework for glass packaging is designed to guarantee product safety while guiding the transition towards more circular and low-carbon manufacturing. For container glass, well-established food-contact and pharmaceutical requirements define composition ranges, leaching limits, and hydrolytic resistance, whereas emerging rules on recycling performance, emission control, and carbon-footprint reporting address the environmental dimension of the value chain. Similar principles are progressively extending to functional and electronic encapsulation, where hermeticity, dielectric integrity, and material traceability must be demonstrated in parallel with circularity and decarbonisation objectives.

**Figure 13:**
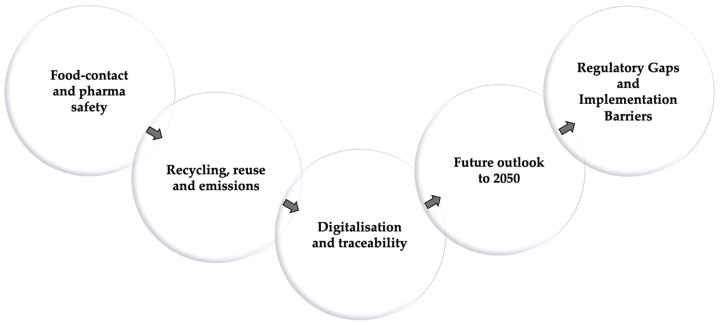
Regulatory and innovation roadmap for packaging glass. Schematic overview of the five thematic domains discussed in Section 5: food-contact and pharmaceutical safety, recycling, reuse and emissions, digitalisation and traceability, future outlook to 2050, and regulatory gaps and implementation barriers, outlining how regulatory practice and innovation pathways jointly shape the evolution of packaging-glass systems.

The discussion in this section follows the sequence summarized in Figure 13: it proceeds from food-contact and pharma safety (Section 5.1) to standards for recycling, reuse and emissions (Section 5.2), then considers digitalisation and traceability tools (Section 5.3), outlines the future outlook to 2050 (Section 5.4), and finally identifies regulatory gaps and implementation barriers that remain to be addressed (Section 5.5).

### 5.1. Food-Contact and Pharmaceutical Compliance

This subsection summarizes the regulatory criteria that determine when a glass container may be safely used in contact with food, beverages, or pharmaceutical formulations. These criteria revolve around three complementary aspects—migration limits, hydrolytic resistance, and compositional purity—which together define the safety benchmarks applied throughout the regulatory framework.

In Europe, the overarching legal requirement is established by Regulation (EC) No 1935/2004 [122], which states that any material intended to contact food must not release substances in quantities that could endanger human health or modify the organoleptic or chemical characteristics of the product. This regulation defines the safety objective; the technical verification of glass performance is instead delegated to specific standards and pharmacopeial tests.

For glass containers, performance is assessed through harmonized technical criteria:

- Hydrolytic resistance is classified through ISO 4802 [123] and EN 1183 [124], which specify the leaching tests, titration procedures, and acceptance limits used to distinguish Type I, II, and III containers.
- Migration behaviour for pharmaceutical use is evaluated according to the European Pharmacopoeia, Section 3.2.1 (Glass containers for pharmaceutical use) [125] and USP <660> (Containers—Glass) [75]. These chapters define limits for pH change, extractable alkalinity, and specific ion release after autoclaving or sterilization, as well as the procedures for surface and whole-container tests.
- Compositional restrictions are included in the same pharmacopeial chapters, which list allowable glass families (borosilicate, soda–lime, aluminosilicate), specify acceptable oxide systems, and prohibit certain toxic elements—such as Pb, Cd, or As—in primary packaging for parenteral preparations.

For high-risk pharmaceutical applications, borosilicate and aluminosilicate glasses routinely meet Type I hydrolytic resistance, with ion release values typically below 0.01 mg Na_2_O-equivalent per litre even after repeated sterilization [9,63]. Recent analytical studies confirm that migration levels are commonly two orders of magnitude lower than the limits imposed by the U.S. FDA and the European Pharmacopoeia, supporting the large safety margin associated with these compositions [54,126].

A separate point concerns the use of recycled glass. Unlike recycled plastics—which are regulated under Commission Regulation (EU) 2022/1616 [127] for recycled plastic materials and articles intended to come into contact with food—cullet for glass packaging is not subject to a dedicated EU framework on decontamination or traceability. Its suitability is instead demonstrated through closed-loop recycling, industrial specifications on cullet purity, and compliance with the hydrolytic and compositional criteria defined in ISO, EN, and pharmacopeial standards.

Taken together, these provisions define the conditions under which glass can be safely employed in sensitive food and pharmaceutical contexts. They provide readers with the essential reference points—migration limits, hydrolytic resistance classes, compositional purity thresholds and the current approaches to managing recycled cullet streams—that underpin the broader regulatory and sustainability discussion developed in the following subsections.

### 5.2. Standards for Recycling, Reuse, and Emission Control

The performance of container glass in recycling, reuse, and emission control is governed by a set of harmonized CEN, ISO, and EU instruments. These frameworks define how recycled content is certified, how recyclability is assessed, and how furnace emissions must be monitored and reported.

At the European level, CEN/TC 261 and the International Commission on Glass (ICG) establish the technical criteria for recyclability, recycled-content verification, and furnace-performance indicators. Standards such as EN 13430 [128] specify when a packaging material can be considered recyclable within existing collection and reprocessing systems, while ISO 14067 [129] provides the methodology for determining the carbon footprint of glass packaging. Although EN 13432 [130] applies specifically to compostable materials, it is frequently used as a comparative benchmark in LCA studies when assessing alternative end-of-life scenarios.

The regulatory framework is reinforced by the Packaging and Packaging Waste Regulation [131], which requires EU Member States to achieve a minimum 80% recycling rate for glass by 2030 and promotes closed-loop circulation through deposit-return systems and extended producer-responsibility schemes. Compliance with these obligations must be demonstrated through mass-balance documentation and Life-Cycle Inventories prepared under ISO 14040/44 [132], ensuring transparent reporting of recycled content and environmental performance [15,21].

Process-related emission control is defined in the Best Available Techniques (BAT) Reference Document for the Glass Industry [133], which specifies performance benchmarks for NO_x_, SO_x_, particulate matter, and energy efficiency. Technologies such as low-NO_x_ oxy-fuel combustion and hybrid-electric melting already meet BAT-associated emission levels, typically achieving 25–30% reductions in combustion-related emissions, consistent with the decarbonisation trajectory of the EU Emissions Trading System [10,121].

Taken together, these standards form the regulatory backbone for evaluating the circularity, environmental performance, and compliance obligations of container glass. They provide measurable criteria—recyclability certification, carbon-footprint reporting, recycling targets, and BAT emission thresholds—that underpin the broader sustainability assessments discussed in the following sections.

### 5.3. Digitalization and Traceability

The ongoing digitalisation of glass manufacturing supports both process optimization and regulatory compliance. Under the EU Sustainable Products Initiative [134], digital product passports (DPPs) will enable batch-level identification of cullet origin, composition and energy source, improving transparency along the value chain.

Real-time monitoring systems that integrate AI-based defect detection, process control, and predictive maintenance increase furnace efficiency while generating detailed records of carbon intensity and quality indicators at batch level [13,121]. These data infrastructures provide the basis for eco-labelling schemes and conformity assessments conducted in accordance with ISO 22095 [135] on chain-of-custody models, linking material traceability, recycled content documentation, and carbon-accounting requirements.

### 5.4. Future Outlook

Future developments in packaging glass are expected to converge along four main directions that integrate decarbonised production, advanced material design, circular logistics and data-driven regulatory compliance. These trajectories reflect both current European policy targets and ongoing industrial innovation, and collectively define the long-term evolution of the glass sector toward 2050.

Decarbonised production will remain the central driver of environmental performance. The expansion of hybrid-electric and hydrogen-assisted melting, supported by renewable-electric boosting and improved refractory materials, is projected to reduce CO_2_ emissions by more than 60% by 2040 in ambitious decarbonisation scenarios [9,21]. Full electrification, when combined with low-carbon electricity, represents the long-term pathway for climate-neutral glass melting.

Advanced compositions and surface engineering are expected to contribute additional reductions in melting temperature and energy demand. Research on ultra-low-alkali borosilicates, rare-earth-modified aluminosilicates, and strengthening strategies—such as ion exchange and thin-film coatings—aims to couple higher mechanical performance with improved melting efficiency while preserving chemical durability [46,52,136].

Integration into circular supply networks will further stabilize closed-loop recycling. High-purity cullet streams, harmonized collection systems, and regionally optimized logistics under the European Circular Economy Action Plan 2.0 are expected to increase recycling rates and reduce the environmental variability associated with transport and sorting (Caspers 2025; Savvova 2025) [56,93]. Deposit-return schemes will play a key role in maintaining the benefits of reuse and conserving material quality.

Digitalisation and data-driven compliance will support transparent carbon accounting and regulatory alignment. Digital product passports [134], batch-level traceability, AI-based monitoring, and standardized chain-of-custody models [135] will link process data, recycled-content verification, and emission reporting, enabling more accurate sustainability assessments and facilitating eco-labelling [13,121].

**Figure 14:**
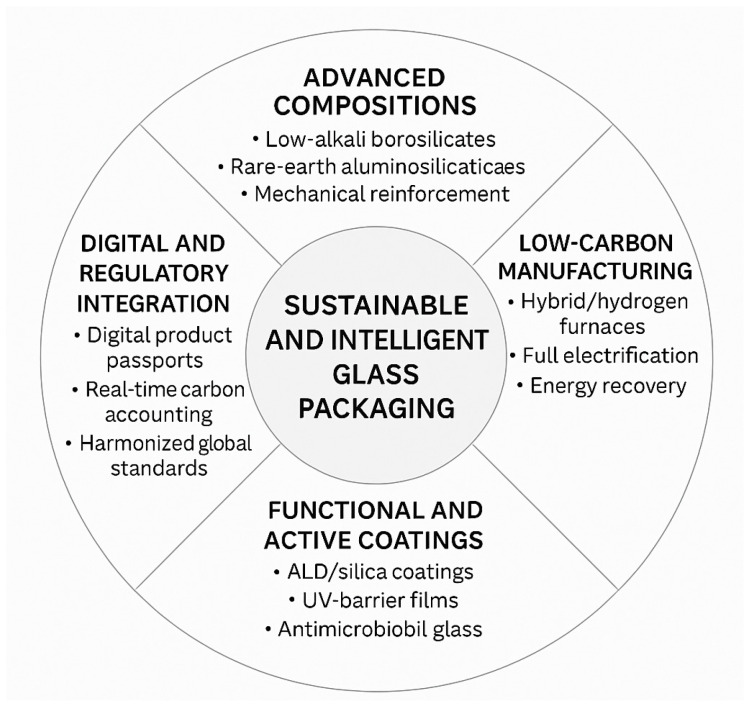
Summary of future directions for packaging glass. Radial diagram summarizing the key innovation domains that will guide the transition toward a sustainable and data-centric glass-packaging ecosystem.

A complementary perspective on long-term innovation is provided by Figure 14, which synthesizes the main directions shaping the future of packaging glass—advanced compositions, low-carbon manufacturing, circular logistics, functional coatings, and regulatory integration.

As Figure 14 indicates, the long-term evolution of packaging glass will depend on the balance between technological maturity, regulatory incentives, and high-quality circularity. These interacting drivers define the framework for evaluating current performance and identifying research priorities across both conventional containers and emerging functional and electronic glass applications.

### 5.5. Regulatory Gaps and Implementation Barriers

Despite the progressive alignment of European policies with circularity and decarbonisation objectives, several regulatory and implementation gaps remain. These affect both container glass and the emerging field of functional and electronic packaging glasses.

A first limitation concerns the uneven performance of national collection and sorting infrastructures. Although PPWR 2025 sets an 80% recycling target by 2030, current cullet streams remain heterogeneous in purity and colour distribution, with significant regional variability [56,93]. This lack of harmonization limits the practical deployment of high-cullet batches, especially in furnaces operating at elevated recycling ratios.

A second gap arises from digital traceability requirements. The Sustainable Products Initiative [134] mandates the introduction of digital product passports with batch-level information on composition, cullet origin, and energy source. However, industrial uptake remains at an early stage, and data integration across sorting plants, furnace operators, and converters is not yet standardized. Full compliance with ISO 22095 chain-of-custody models therefore requires additional investment in monitoring systems, data harmonization, and long-term storage infrastructures [13,121].

Emission-control targets under BREF 2022 also present implementation challenges. The transition to low-NO_x_ oxy-fuel furnaces and hybrid-electric melting requires refractory compatibility, burner redesign, and reliable access to low-carbon electricity. These requirements are only partially met in many industrial plants, delaying the expected reductions in CO_2_ and NO_x_ emissions [10,21].

For functional and electronic packaging glasses, regulatory gaps are more pronounced. No harmonized recycling routes, LCA datasets, or emission factors exist for low-temperature sealing frits, bonding glasses, or dielectric microelectronic substrates [41,113,114]. Broader assessments of electronics and semiconductor supply chains consistently highlight the lack of robust upstream life-cycle inventories for advanced materials, with most datasets incomplete or reliant on proxy assumptions [39,137,138,139,140,141]. As a result, functional-glass systems cannot yet be evaluated using the same quantitative circularity and climate indicators applied to container glass.

These gaps indicate that regulatory alignment and technological readiness must advance in parallel if the glass industry is to achieve the circularity and climate-neutrality objectives targeted for 2040–2050.

The main regulatory instruments affecting packaging glass are summarized in Table 4, which links their key requirements with industrial implications and the current gaps in implementation.

**Strategic Priorities for 2030–2050**

Achieving a climate-neutral and fully circular glass industry requires coordinated progress across regulatory, technological, and infrastructural domains. By 2030, harmonized collection systems, high-purity cullet streams, and the full deployment of digital product passports will be essential to stabilize recycling performance and support transparent carbon accounting, as anticipated under PPWR 2025 and the Sustainable Products Initiative (SPI 2024; Caspers 2025; Savvova 2025) [56,93,134]. Between 2030 and 2040, scaling up hybrid-electric and hydrogen-assisted melting, together with updated emission and carbon-footprint standards, is expected to deliver substantial reductions in operational emissions, building on the decarbonisation trajectories identified for melting furnaces [9,107]. Beyond 2040, digital-twin lifecycle management and real-time material traceability are anticipated to connect regulatory compliance with process optimization, supported by the progressive integration of AI-based monitoring and batch-level carbon accounting in industrial practice [13,121].

For functional and electronic packaging glasses, the strategic priority is the creation of dedicated LCA datasets, harmonized end-of-life pathways and composition-specific guidance. These developments are essential to enable future integration of sealing and dielectric glass systems into standardized circularity and decarbonisation metrics. Achieving this alignment will require coordinated progress in data infrastructure, regulatory frameworks and industrial traceability.

## 6. Discussion

This review compared composition–structure relationships, performance domains and circularity aspects for conventional container glasses, and composition–performance relationships for functional/electronic encapsulation systems.

Across the evidence surveyed (2018–2025), three themes emerge.

First, network design (B_2_O_3_/Al_2_O_3_ and modifier balance) governs the joint evolution of CTE, hydrolytic class, and elastic response; borosilicate and aluminosilicate systems consistently outperform soda–lime glass in thermal–chemical stability at the expense of higher melting temperature. Second, surface engineering (ion exchange and thin films) narrows the mechanical and chemical gap without compromising optical clarity, enabling lightweight designs and extending service life. Third, circular manufacturing (high-purity cullet, hybrid/oxy furnaces, digital control) delivers material and energy efficiencies that dominate life-cycle benefits.

In functional and electronic encapsulation, these same levers operate under tighter constraints, as dielectric loss, CTE-matching requirements, and sealing-temperature limits narrow the compositional design space but enable system-level integration of optical, electronic, and microfluidic functions.

A practical implication is the application-specific matching: soda–lime remains optimal for high-throughput food/beverage lines; borosilicate is unmatched for parenterals and heat-sterilized products; aluminosilicate enables thin-wall premium containers and repeated thermal cycles; high-cullet soda–lime provides the lowest footprint where supply chains support closed-loop logistics. For functional and electronic packaging, tailored borosilicate/aluminosilicate and lead-free sealing glasses are selected for hermeticity, dielectric integrity, and CTE matching to metals or semiconductors, prioritizing interfacial reliability over bulk strength. At the device level, this includes borosilicate caps and glass-to-metal seals for MEMS and implantable components, low-alkali aluminosilicate interposers with through-glass vias for RF and 3D system-in-package architectures, and Bi–B–Zn or phosphate-based frit glasses for low-temperature wafer-level sealing and microfluidic cartridges. In extreme-containment scenarios, corrosion-resistant borosilicates support long-term immobilization.

Methodological limitations of the literature include heterogeneous test protocols (e.g., hydrolytic resistance, fracture statistics), variable reporting of flaw populations after forming, and LCA assumptions that differ in energy mixes and transport scenarios. In functional/electronic applications, additional variability arises from leak-rate methods and dielectric testing under humidity/thermal cycling. Harmonized testing (EN/ISO/USP), shared data schemas, and batch-level traceability would improve cross-study comparability.

Surface-strengthening trade-offs deserve attention: deep ion-exchange layers and ALD films increase reliability but introduce process complexity and potential cost/throughput constraints; long-term stability under repeated washing/sterilization (reuse loops) should be documented with standardized cycling. For encapsulation glasses, the stability of seals and coatings after combined electrical, thermal, and moisture stress requires coordinated protocols that link hermetic leak testing with dielectric breakdown and optical drift.

On recycling, the consensus is robust: each +10% cullet cuts furnace energy ≈3% and CO_2_ proportionally; properties remain within specification after multiple remelts when colour and composition are controlled. Research gaps persist on contaminant tolerance, de-alkalization during repeated loops, and real-time cullet quality grading. For functional/electronic systems, end-of-life pathways and compositional transparency (e.g., dopants, sealing additives) need integration into circularity metrics and design-for-disassembly guidelines. At present, these functional and electronic systems have no established recycling routes, harmonized LCA datasets, or EN/ISO end-of-life standards, so their contribution to circularity remains largely absent from current regulatory and industrial indicators.

Decarbonization pathways are converging—hybrid/electric boosting now, hydrogen assistance mid-term, and full electrification in the long run—yet refractory durability, grid carbon intensity, and CAPEX remain rate-limiting factors. Digital twins and product passports can connect quality, carbon accounting, and compliance. These tools also enable traceability in functional/electronic glass packaging, where verified material data must be linked to hermetic and dielectric reliability.

Finally, future research should integrate multiscale modelling (defect-controlled strength), operando corrosion analytics, and techno-economic LCA that couples furnace technology with regional cullet logistics, to objectively guide the selection of “best-fit” glass families for each packaging segment. Extending these approaches to functional and electronic encapsulation—through standardized hermetic/dielectric testing, CTE-matched seal design, and circular end-of-life strategies—will consolidate glass as a platform bridging conventional containers and advanced multifunctional packaging. This is particularly relevant for high-value, low-volume sectors such as microelectronics, photonics, and biomedical diagnostics, where glass functions simultaneously as a structural, optical, and dielectric platform.

## 7. Conclusions and Perspectives

The evolution of packaging glass demonstrates a rare combination of material resilience, scientific refinement, and regulatory alignment. From traditional soda–lime systems to advanced aluminosilicate and borosilicate compositions, continuous optimization of the glass network has progressively enhanced mechanical strength, chemical stability, and thermal endurance while maintaining the complete recyclability that distinguishes glass from all other packaging materials.

Recent developments in manufacturing—hybrid-electric and oxy-fuel furnaces, digital process control, and closed-loop recycling—have substantially reduced energy demand and CO_2_ emissions, supporting the global transition toward low-carbon production. Life-cycle assessments confirm that each 10% increase in cullet content lowers the environmental footprint proportionally, and modern collection systems now enable recycling rates above 75%. These results place packaging glass at the core of the European Green Deal strategy for circular materials.

Beyond conventional containers, functional and electronic glass packaging relies on similar principles of composition engineering and process control, but its sustainability frameworks are still emerging. While advances in digital monitoring and high-integrity sealing support reliability and traceability, established pathways for recycling, end-of-life management and LCA validation are not yet available for these systems. In these domains, circular-design strategies remain prospective rather than operational, indicating a critical area for future standardization and materials development.

Future advances are expected to emerge from the convergence of composition engineering, functional coatings, and digital traceability. Low-alkali and rare-earth-modified glasses will offer improved mechanical and thermal performance at lower melting temperatures, while nanometric coatings based on silica or alumina will further enhance UV shielding, antimicrobial protection, and surface durability. At the same time, digital product passports, real-time emission monitoring, and AI-driven quality control will guarantee full transparency and compliance across the supply chain, connecting production, regulation, and sustainability metrics in a single verified data ecosystem.

Looking forward, the synergy between material innovation, decarbonized processes, and circular logistics will drive glass manufacturing toward climate-neutral, intelligent, and multifunctional production by 2050. The integration of scientific insight with digital and regulatory frameworks will secure glass’s role as the benchmark for safety, recyclability, and long-term environmental stewardship—bridging traditional packaging and advanced functional encapsulation within a unified circular economy framework.

## Figures and Tables

**Table 4:** Regulatory requirements, industrial implications, and current gaps for packaging glass.

Regulation /Standard	Key Requirement	Industrial Implication	Current Gap
PPWR 2025 [131]	≥80% glass recycling by 2030; promotion of reuse and DRS schemes	Need for harmonized collection, colour sorting, high-purity cullet streams	Strong regional variability in collection rates; inconsistent cullet quality
SPI 2024/Digital Product Passport [134]	Batch-level traceability of cullet origin, composition, and energy source	Integration of AI monitoring, data-sharing platforms, ISO 22095 compliance	Limited industrial deployment; lack of unified traceability infrastructure
BREF 2022 (BAT) [133]	Low-NO_x_ and energy-efficient melting	Hybrid/oxy-fuel furnaces, improved insulation, heat recovery	Electrical grid capacity, refractory constraints, high investment thresholds
ISO 14040/44 and ISO 14067 [129,132]	Transparent LCA and carbon-footprint reporting	Standardized CO_2_ accounting; certification of low-carbon batches	Lack of harmonized datasets for certain compositions; no LCA data for functional glasses
EC 1935/2004; EN 1183; ISO 4802; USP <660> [75,122,123,124]	Migration limits and hydrolytic resistance for food/pharma use	Material validation, compositional control, surface-quality assurance	Not applicable to functional/electronic glasses; no parallel standards

## Data Availability

No new data were created or analyzed in this study. Data sharing is not applicable to this article.

## Abbreviations

The following abbreviations are used in this manuscript:

AFM	Atomic Force Microscopy
AI	Artificial Intelligence
ALD	Atomic Layer Deposition
BAT	Best Available Techniques
BREF	Best Available Techniques Reference Document
CAPEX	Capital Expenditure
CEN	European Committee for Standardization
CO_2_e	Carbon Dioxide Equivalent
CTE	Coefficient of Thermal Expansion
DFT	Density Functional Theory
DPP	Digital Product Passport
DRS	Deposit Return Scheme
EPR	Extended Producer Responsibility
ESPR	Ecodesign for Sustainable Products Regulation
EU	European Union
FDA	Food and Drug Administration
FBG	Fibre Bragg Grating
FEVE	European Container Glass Federation
FTIR	Fourier Transform Infrared Spectroscopy
GMP	Good Manufacturing Practice
GWP	Global Warming Potential
HPFS	High-Purity Fused Silica
HTOL	High-Temperature Operating Life
ICP-MS	Inductively Coupled Plasma Mass Spectrometry
ICG	International Commission on Glass
IR	Infrared
ISO	International Organization for Standardization
LCA	Life Cycle Assessment
LAS	Lithium Aluminosilicate
MEMS	Micro-Electro-Mechanical System
MOEMS	Micro-Opto-Electro-Mechanical System
NNPB	Narrow-Neck Press-and-Blow
Ph. Eur.	European Pharmacopoeia
PPWD	Packaging and Packaging Waste Directive
PPWR	Packaging and Packaging Waste Regulation (proposal)
PRISMA	Preferred Reporting Items for Systematic Reviews and Meta-Analyses
PV	Photovoltaic
REACH	Registration, Evaluation, Authorisation and Restriction of Chemicals
RF	Radio Frequency
RoHS	Restriction of Hazardous Substances
SDGs	Sustainable Development Goals
SPI	Sustainable Products Initiative
TGV	Through-Glass Via
ToF-SIMS	Time-of-Flight Secondary Ion Mass Spectrometry
TOC	Total Organic Carbon
UN	United Nations
USP	United States Pharmacopeia
UV	Ultraviolet
XPS	X-ray Photoelectron Spectroscopy
